# Supplementary material for: Identification of pyrogallol as a warhead in design of covalent inhibitors for the SARS-CoV-2 3CL protease
Source: Nat Commun. 2021 Jun 15;12:3623. doi: 10.1038/s41467-021-23751-3 (PMC8206078; doi:10.1038/s41467-021-23751-3)
Supplement: Supplementary file 1 — Supplementary Information [file 41467_2021_23751_MOESM1_ESM.pdf]

Supplementary Information

Identification of pyrogallol as a warhead in design of covalent inhibitors for the SARS-CoV-2 3CL protease

Haixia Su[1,2,9], Sheng Yao[2,3,9], Wenfeng Zhao[1,9], Yumin Zhang[4,9], Jia Liu[2,3,9], Qiang Shao[1,9], Qingxing Wang[2,4], Minjun Li[5], Hang Xie[1], Weijuan Shang[4], Changqiang Ke[3], Lu Feng[3], Xiangrui Jiang[1,2], Jingshan Shen[1,2], Gengfu Xiao[2,4], Hualiang Jiang[1,2,6,8], Leike Zhang[2,4,10*] Yang Ye[2,3,7,10*], Yechun Xu[1,2,8,10*]

[1]CAS Key Laboratory of Receptor Research, and Drug Discovery and Design Center, Shanghai Institute of Materia Medica, Chinese Academy of Sciences, Shanghai 201203, China

[2]University of Chinese Academy of Sciences, Beijing 100049, China

[3]State Key Laboratory of Drug Research, and Natural Products Chemistry Department, Shanghai Institute of Materia Medica, Chinese Academy of Sciences, Shanghai 201203, China

[4]State Key Laboratory of Virology, Wuhan Institute of Virology, Center for Biosafety Mega-Science, Chinese Academy of Sciences, 430071 Wuhan, China

[5]Shanghai Synchrotron Radiation Facility, Shanghai Advanced Research Institute, Chinese Academy of Sciences, Shanghai 201210, China

[6]Shanghai Institute for Advanced Immunochemical Studies and School of Life Science and Technology, ShanghaiTech University, Shanghai 201210, China

[7]School of Life Science and Technology, ShanghaiTech University, Shanghai 201210, China

[8]School of Pharmaceutical Science and Technology, Hangzhou Institute for Advanced Study, University of Chinese Academy of Sciences, Hangzhou 310024, China.

[9]These authors contributed equally: Haixia Su, Sheng Yao, Wenfeng Zhao, Yumin Zhang, Jia Liu and Qiang Shao

[10]These authors jointly supervised this work: Leike Zhang, Yang Ye, Yechun Xu.

[*]correspondence: zhangleike@wh.iov.cn; yye@simm.ac.cn; ycxu@simm.ac.cn.

Table of Contents

I. Supplementary Methods....5

General methods 5

General procedures for synthesis of myricetin and dihydromyticetin derivatives..... 5

II. Supplementary Figures 10

Supplementary Fig. 1 10

Supplementary Fig. 2 11

Supplementary Fig. 3 12

Supplementary Fig.4 13

Supplementary Fig. 5 14

Supplementary Fig. 6 15

Supplementary Fig. 7 16

Supplementary Fig. 9 18

Supplementary Fig. 10 19

Supplementary Fig. 11 20

Supplementary Fig. 12 21

III. Supplementary Tables 22

Supplementary Table 1 22

Supplementary Table 2 25

Supplementary Table 3 26

Supplementary Table 4 27

Supplementary Table 5 29

Supplementary Table 6 30

Supplementary Table 7 31

Supplementary Table 8 32

III. Spectra of myricetin derivatives 33

I. Supplementary Methods

General methods

Optical rotations were obtained on a Rudolph Research Analytical Autopol VI 90079 polarimeter (Hackettstown, NJ). Analytical HPLC and ESIMS spectra were performed on a Waters 2695 instrument with a 2998 PDA detector coupled with a Waters Acquity ELSD and a Waters 3100 SQDMS detector using a Waters Sunfire RP C18 column (4.6 × 150 mm, 5 μm) with a 1.0 mL/min flow rate. Preparative HPLC was performed on a Waters 2545 Binary Gradient Module instrument with a Waters 2489 UV/Visible Detector using a Waters Sunfire Prep C18 column (5 μm, 30 × 150 mm). HRESIMS data were recorded on a Waters Synapt G2-Si Q-Tof mass detector. 1H and 13C NMR spectra were recorded on a Bruker AVANCE III 600 MHz instrument. Chemical shifts were reported in ppm (δ) coupling constants (J) in hertz. Chemical shifts are reported in ppm units with Me4Si as a reference standard.

General procedures for synthesis of myricetin and dihydromyticetin derivatives

7-*O*-methyl-myricetin (3). Myricetin (1.0 mmol, 318 mg) was dissolved in acetone (25 mL) in a 50 mL of flask. To this solution Me_2SO_4 (1.0 mmol, 95 μL, 1.0 eq.) and K_2CO_3 (1.0 mmol, 138 mg, 1.0 eq.) were added. The reaction mixture was stirred at 60 °C for 1.5 h before quenched by water. The mixture was suspended in 180 mL water containing 2 mL of 2N HCl and extracted with ethyl acetate (50 mL × 3). The combined organic layer was washed with water and brine, then dried with $MgSO_4$. The residue was subjected to preparative HPLC (MeCN in water containing 0.1% HCOOH, 20-50%, 0-25 min) to obtain 7-*O*-Methyl-myricetin (**3**) as a yellow solid (25 mg, yield: 7.5%). Purity: 96.31% (360 nM), 96.01% (260 nm); ESI-MS *m/z* 333.19 $[M+H]^+$, 331.07 $[M-H]^-$; ^1H NMR (DMSO-d_6, 500 MHz) δ 12.50 (s, 1H, 5-OH), 7.28 (s, 2H, H-2', 6'), 6.65 and 6.35 (d, J = 2.2 Hz, each 1H, H-5, 7), 3.87 (s, 3H,7-OC$\underline{H}_3$); ^{13}C NMR (DMSO-d_6, 125 MHz) δ 175.8 (C-4), 164.8 (C-7), 160.4 (C-5), 156.0 (C-8a), 147.3 (C-4'), 145.7 (C-3',5'), 136.1 (C-3), 136.0 (C-1), 120.7 (C-1'), 107.3 (C-2',6'), 103.9 (C-4a), 97.4 (C-6), 91.7 (C-8), 56.0 (7-O$\underline{C}H_3$); HRESI-MS (*m/z*): $[M-H]^-$ calcd. for

$C_{16}H_{11}O_8$, 331.0454; found 331.0452.

7-*O*-ethyl-myricetin (4). Following the procedure described for preparation of compound **3**, compound **4** was prepared from myricetin and Et_2SO_4 as a yellow solid (55 mg, yield: 16%). Purity: 99.83% (360 nm), 99.54% (260 nm); ESI-MS *m/z* 347.19 $[M+H]^+$, 345.06 $[M-H]^-$; ^1H NMR (DMSO-d_6, 500 MHz) δ 12.48 (s, 1H, 5-OH), 7.27 (s, 2H, H-2', 6'), 6.62 and 6.32 (d, J = 2.2 Hz, each 1H, H-5, 7), 4.15 (q, J = 7.0 Hz, 2H, 7-OC$\underline{H}_2$CH$_3$), 1.35 (t, J = 7.0 Hz, 3H, 7-OCH$_2$C$\underline{H}_3$); ^{13}C NMR (DMSO-d_6, 125 MHz) δ 175.8 (C-4), 164.1 (C-7), 160.4 (C-5), 156.0 (C-8a), 147.3 (C-4'), 145.7 (C-3',5'), 136.1 (C-3), 136.0 (C-1), 120.7 (C-1'), 1107.3 (C-2',6'), 103.9 (C-4a), 97.7 (C-6), 92.1 (C-8), 64.1 (7-O$\underline{C}$H$_2$CH$_3$), 14.4 (7-OCH$_2$$\underline{C}H_3$); HRESI-MS (*m/z*): $[M-H]^-$ calcd. for $C_{17}H_{13}O_8$, 345.0610; found 345.0606.

7-*O*-isoamyl-myricetin (**5**). Myricetin (1.0 mmol, 318 mg) was dissolved in anhydrous DMF (10 mL) in a 25 mL of flask. To this solution isoamyl bromide (1.0 mmol, 125.8 μL, 1.0 eq.) and K_2CO_3 (1.0 mmol, 138 mg, 1.0 eq.) were added. The reaction mixture was stirred for 90 min at room temperature before filtration. The filtrate was diluted with ethyl acetate (100 mL) and then washed with water, and then brine. The organic layer was dried with $MgSO_4$ and then evaporated. The residue was subjected to preparative HPLC (MeCN in water containing 0.1% HCOOH, 40-60%, 0-25 min) to get **5** as a yellow solid (57 mg, yield: 15%). Purity: 99.94% (360 nm), 99.83% (260 nm); ESI-MS *m/z* 389.27 $[M+H]^+$, 387.15 $[M-H]^-$; ^1H NMR (Acetone-d_6, 500 MHz) δ 12.10 (s, 1H, 5-OH), 7.44 (s, 2H, H-2', 6'), 6.67 and 6.30 (d, J = 2.2 Hz, each 1H, H-5, 7), 4.18 (t, J = 6.7 Hz, 2H, 7-OC$\underline{H}_2$), 1.86 (m, 1H, -CH-), 1.72 (dd, J = 6.7 and 6.8 Hz, 2H, 7-OCH$_2$C$\underline{H}_2^-$), 0.99 (d, J = 6.7 Hz, 6H, -CH$_3$ × 2); ^{13}C NMR (Acetone-d_6, 125 MHz) δ 176.5 (C-4), 166.1 (C-7), 161.8 (C-5), 157.6 (C-8a), 147.2 (C-4'), 146.4 (C-3',5'), 137.1 (C-3), 136.4 (C-1), 122.7 (C-1'), 108.4 (C-2',6'), 104.7 (C-4a), 98.7 (C-6), 93.1 (C-8), 68.0 (7-O$\underline{C}$H$_2$-), 38.4 (7-OCH$_2$$\underline{C}H_2^-$), 25.8 (-CH-), 22.8 (-CH$_3$× 2); HRESI-MS (*m/z*): $[M-H]^-$ calcd. for $C_{20}H_{19}O_8$, 387.1080; found 387.1074.

7-*O*-cyclopentylmethyl-myricetin (**6**). Following the procedure described for preparation of compound **5**, compound **6** was prepared from myricetin and cyclopentyl bromide as a yellow solid (64 mg, yield: 16%). Purity: 99.69% (360 nm), 99.12% (260

nm); ESI-MS *m/z* 401.28 $[M+H]^+$, 399.17 $[M-H]^-$; ^1H NMR (Acetone-d_6, 600 MHz) δ 12.09 (s, 1H, 5-OH), 7.44 (s, 2H, H-2', 6'), 6.67 and 6.31 (d, J = 2.1 Hz, each 1H, H-5, 7), 4.03 (d, J = 7.0 Hz, 2H, 7-OC$\underline{H}_2$-), 2.41 (m, 1H, -CH-), 1.85, 1.88, 1.60 and 1.41 (m, each 2H, -CH_2- in cyclopentane); ^{13}C NMR (Acetone-d_6, 150 MHz) δ 176.5 (C-4), 166.2 (C-7), 161.8 (C-5), 157.7 (C-8a), 147.2 (C-4'), 146.4 (C-3',5'), 137.1 (C-3), 136.5 (C-1), 122.7 (C-1'), 108.4 (C-2',6'), 104.7 (C-4a), 98.7 (C-6), 93.1 (C-8), 73.6 (7-O$\underline{C}H_2$-), 39.7 (7-$OCH_2\underline{C}H^-$), 30.0, 30.0, 26.0, 26.0 (-$\underline{C}H_2$-×4 in cyclopentane); HRESI-MS (*m/z*): $[M-H]^-$ calcd. for $C_{20}H_{19}O_8$, 399.1080; found 399.1074.

7-*O*-methyl-dihydromyricetin (7). Following the procedure described for preparation of compound **3**, compound **7** was prepared from dihydromyricetin as a white solid (27 mg, yield: 8%). $[\alpha]_D^{20}$ + 6.8 (*c* 1.0, in MeOH); ee%: +39%; Purity: 99.95% (290 nm); ESI-MS *m/z* 335.47 $[M+H]^+$, 333.09 $[M-H]^-$; ^1H NMR (Acetone-d_6, 600 MHz) δ 11.69 (s, 1H, 5-OH), 6.63 (s, 2H, H-2', 6'), 6.07 and 6.05 (d, J = 2.2 Hz, each 1H, H-5, 7), 4.98 and 4.59 (d, J = 11.4 Hz, each 1H, H-2,3), 3.86 (s, 3H, 7-OCH_3); ^{13}C NMR (Acetone-d_6, 150 MHz) δ 198.5 (C-4), 169.2 (C-7), 164.7 (C-8a), 163.9 (C-5), 146.3 (C-3',5'), 134.2 (C-4'), 134.1 (C-3), 136.5 (C-1), 129.0 (C-1'), 108.0 (C-2',6'), 102.1 (C-4a), 95.7 (C-8), 94.7 (C-6), 84.8 (C-2), 73.3 (C-3), 56.4 (7-O$\underline{C}H_3$); HRESI-MS (*m/z*): $[M-H]^-$ calcd. for $C_{16}H_{13}O_8$, 333.0610; found 333.0609.

Myricetin-7-yl 5,5-dimethyl-1,3,2-dioxayl phosphate (8). Myricetin (1.0 mmol, 318 mg) and triethylamine (1.05 mmol, 146 μL, 1.05 equiv.) were dissolved in anhydrous DMF (10 mL) in a 25 mL of flask. This solution was added by 2-Chloro-5,5-dimethyl-1,3,2-dioxaphosphorinane 2-oxide (1.05 mmol, 194 mg, 1.05 equiv.) dropwise at 0 °C. The reaction mixture was stirred for 3 h at room temperature, and then filtered through celite to remove NEt3.HCl salt. The filtrate was evaporated and the resulting residue was subjected to Auto-preparative HPLC (MeCN in water containing 0.1% HCOOH, 30-50%, 0-35 min) to get **8** as a yellow solid (100 mg, 21%). Purity: 99.98% (360 nm), 100% (260 nm); ESI-MS *m/z* 467.22 $[M+H]^+$, 465.10 $[M-H]^-$; ^1H NMR (DMSO-d_6, 500 MHz) δ 12.07 (s, 1H, 5-OH), 7.30 (s, 2H, H-2', 6'), 7.05 and 6.25 (d, J = 3.1 Hz, each 1H, H-5, 7), 4.35 (d, J = 10.6 Hz, 2H, OCH_2-), 4.07 (dd, J = 10.8 and 10.6 Hz, 2H, OCH_2-), 1.20 and 0.87 (s, each 3H, CH_3 × 2); ^{13}C NMR

(DMSO-d_6, 125 MHz) δ 176.5, 161.1, 155.4, 155.0 (154.9), 148.8, 146.3, 146.3, 137.2, 136.9, 120.8, 107.9, 107.9, 107.3, 102.3 (102.2), 98.9 (98.8), 79.0, 78.9, 32.4, 21.4, 19.5; ^{31}P NMR (DMSO-d_6, 125 MHz) δ -14.6; HRESI-MS (*m/z*): [M-H]$^-$ calcd. for $C_{20}H_{18}O_{11}P$, 465.0587; found 465.0588.

Myricetin-7-yl diphenyl phosphate (9). Following the procedure described for preparation of compound **8**, compound **9** was prepared from myricetin as a yellow solid (100 mg, yield: 18%). Purity: 99.37% (360 nm), 99.28% (260 nm); ESI-MS *m/z* 551.24 [M+H]$^+$, 549.12 [M-H]$^-$; ^{1}H NMR (Acetone-d_6, 500 MHz) δ 12.37 (s, 1H, 5-OH), 7.47 (d, J = 7.5 Hz, 4H, H-1a,6a × 2),7.45 (s, 2H, H-2', 6'), 7.35 (dd, J = 7.5 and 1.1 Hz, 4H, H-2a,5a × 2), 7.30 (dt, J = 7.5 and 1.1 Hz, 2H, H-3a × 2), 7.10 and 6.67 (d, J = 1.3 Hz, each 1H, H-5, 7); ^{13}C NMR (Acetone-d_6, 125 MHz) δ 176.0, 161.3, 155.7, 155.2 (155.1), 150.0, 150.0, 147.9, 145.7, 145.7, 136.8, 136.1, 120.8, 130.1, 130.1, 129.1, 129.1, 126.0, 123.3, 120.3, 120.2, 120.1, 120.1, 107.9, 107.9, 107.1, 102.0 (102.0), 99.1 (99.0); ^{31}P NMR (Acetone-d_6, 125 MHz) δ -18.5; HRESI-MS (*m/z*): [M-H]$^-$ calcd. for $C_{27}H_{18}O_{11}P$, 549.0587; found 549.0591.

Dihydromyricetin-7-yl diphenyl phosphate (10). Following the procedure described for preparation of compound **8**, compound **10** was prepared from dihydromyricetin as a yellow solid (148 mg, yield: 27%). $[\alpha]_D^{20}$ + 9.0 (*c* 1.0, in MeOH); ee%: +28%; Purity: 99.78% (330 nm), 99.55% (280 nm); ESI-MS *m/z* 553.26 [M+H]$^+$, 551.13 [M-H]$^-$; ^{1}H NMR (Acetone-d_6, 500 MHz) δ 11.69 (s, 1H, 5-OH), 7.46 (d, J = 7.5 Hz, 4H, H-1a,6a × 2),7.34 (s, 2H, H-2', 6'), 7.34 (dd, J = 7.5 and 1.1 Hz, 4H, H-2a,5a × 2), 7.30 (dt, J = 7.5 and 1.1 Hz, 2H, H-3a × 2), 6.64 (s, 2H, H-2',6'), 6.46 and 6.44 (d, J = 2.1 Hz, each 1H, H-5, 7), 5.10 and 4.73 (d, J = 11.6 Hz, each 1H, H-2, H-3); ^{13}C NMR (Acetone-d_6, 125 MHz) δ 200.0, 164.2, 163.8, 158.6(158.5), 151.3(151.2), 146.4, 134.4, 131.0, 131.0, 131.0, 131.0,128.5, 126.9, 126.9, 121.0,121.0,120.9, 120.9, 108.1, 108.1, 105.4, 101.7 (101.6), 100.6 (100.5), 85.0, 73.4; ^{31}P NMR (Acetone-d_6, 125 MHz) δ -18.7; HRESI-MS (*m/z*): [M-H]$^-$ calcd. for $C_{27}H_{20}O_{11}P$, 551.0743; found 551.0740.

Myricetin-GSH adduct. Myricetin (1.0 mmol, 318 mg) and glutathione (25 mmol, 7.68 g, 25 equiv.) were dissolved in 300 mL of phosphate buffer solution (10 mM, pH

7.4) in a 500 mL of flask. The reaction mixture was incubated at room temperature overnight. The reaction mixture was extracted with ethyl acetate (100 mL × 3) and the combined organic layer was dried with anhydrous MgSO4, evaporated under reduced pressure. The resulting residue was subjected to Auto-preparative HPLC (MeCN in water containing 0.1% HCOOH, 5-25%, 0-35 min) to get myricetin-GSH adduct as a yellow solid (78 mg, 13%). Purity: 99.58% (340 nm), 99.61% (250 nm); ESI-MS *m/z* 624.14 $[M+H]^+$, 622.17 $[M-H]^-$; ^{1}H NMR (DMSO-d_6, 500 MHz) δ 12.48 (s, 1H, 5-OH), 8.24 (brs, 2H, $-NH_2$), 6.50 (s, 1H, H-6'), 6.43 (brs, 1H, H-7), 6.15 (d, J = 1.7 Hz, 1H, H-7), 4.24 (dd, J = 11.9, 5.7 Hz, 1H, H-4"), 3.62 (brs, 2H, H-2"), 3.36 (t, J = 6.7 Hz, 1H, H-8"), 3.06 and 2.91 (dd, J = 13.2, 5.6 Hz, each 1H, H-10"a, 10"b), 2.19-2.28 (m, 2H, H-6"), 1.85-1.92 (m, 2H, H-7"); ^{13}C NMR (DMSO-d_6, 125 MHz) δ 176.3, 171.8, 171.0, 170.6, 164.6, 160.8, 156.5, 150.0, 148.3, 146.5, 136.6, 135.2, 126.0, 110.0, 109.7, 103.4, 98.5, 93.8, 53.2, 52.6, 41.3, 37.3, 31.5, 26.7; HRESI-MS (*m/z*): $[M+H]^+$ calcd. for $C_{25}H_{26}N_3O_{14}S$, 624.1135; found 624.1132

II. Supplementary Figures

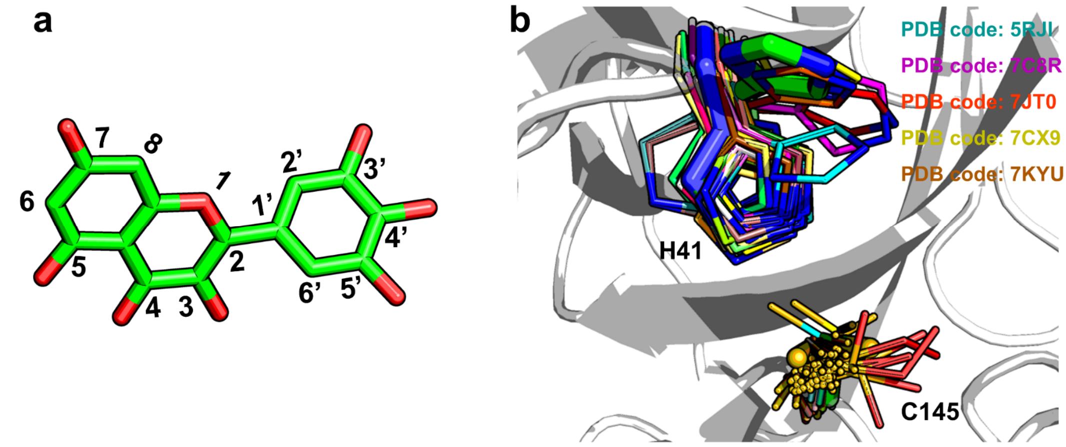

Supplementary Fig. 1 a, Structure of myricetin. **b**, Superimposition of a total of 306 structures of the SARS-CoV-2 $3CL^{pro}$ available in Protein Data Bank on April 1, 2021, including the structure of SARS-CoV-2 $3CL^{pro}$ in complex with myricetin. Two catalytic residues, His41 and Cys145 in the structures bound with myricetin and baicalein were highlighted by green and blue sticks, respectively, while they were shown as lines in other structures. The SARS-CoV-2 $3CL^{pro}$ is shown as cartoon. The side chain of His41 (green sticks) in the SARS-CoV-2 $3CL^{pro}$/myricetin structure adopted a different orientation, compared to it did in most reported structures, to form π-π interactions with myricetin. Likewise, His41 in another five complex structures (PDB codes: 5RJI, 7C8R, 7JT0, 7CX9, and 7KYU) also rotated to form H-bond or π-π interactions with the bound ligands.

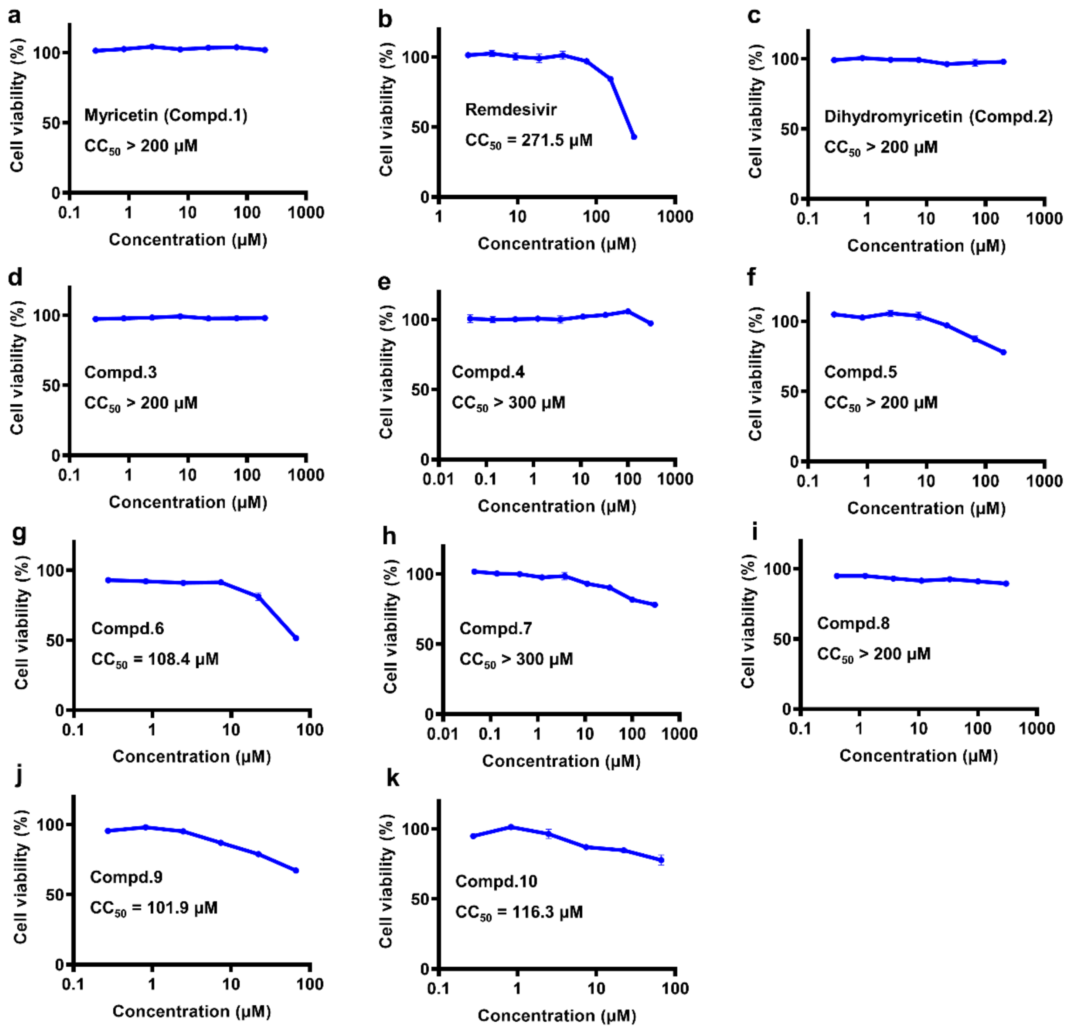

Supplementary Fig. 2 Cytotoxicities of myricetin, remdesivir and myricetin derivatives in Vero E6 cells. Error bars represent mean ± SD of three independent experiments.

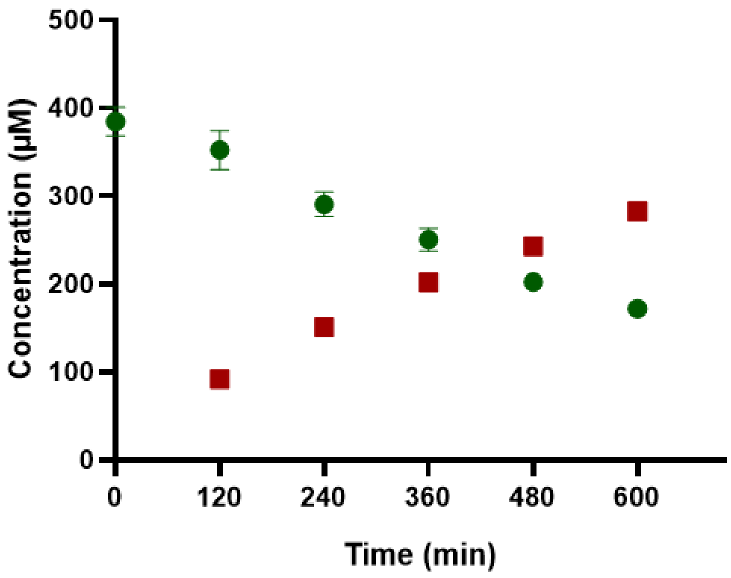

Supplementary Fig. 3 The concentration of myricetin (dark red) and myricetin-GSH covalent adduct (green) detected by MS. The myricetin decreased and the adduct increased with the increase of reaction time. Error bars represent mean ± SD of three independent experiments

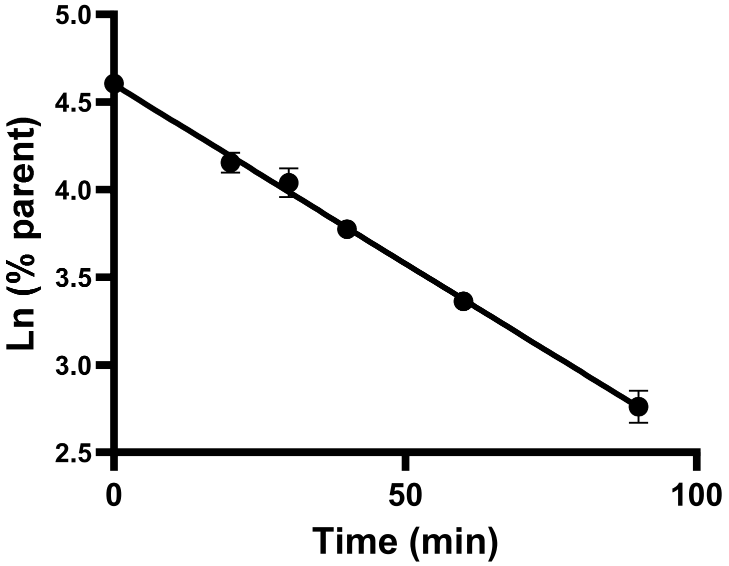

Supplementary Fig.4 Half-life time determination of N-Phenylacylamide reacting with glutathione. N-Phenylacylamide (400 μM at a final concentration) was incubated with 10 mM GSH for 20, 30, 40, 60, and 90 min, respectively. The remaining N-phenylacylamide was determined by HPLC/MS. Ln (%parent) was plotted against the incubation time using GraphPad Prism software 8.0 (GraphPad Software, Inc., San Diego,CA, USA), where %parent means the percentage of the remaining N-phenylacylamide at different incubation time. Error bars represent mean ± SD of three independent experiments

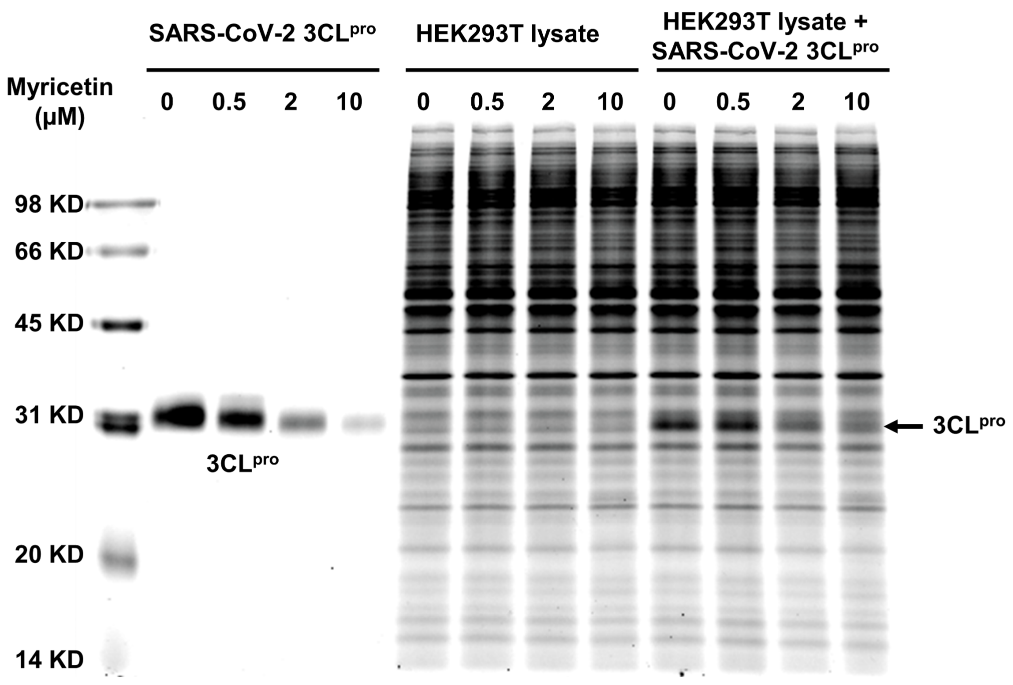

Supplementary Fig. 5 Selectivity evaluation of myricetin in HEK293T lysate by the gel-based competitive ABPP. Recombinant SARS-CoV-2 $3CL^{pro}$ at a final concentration of 1 μg/mL (the first four samples), the HEK293T lysate at a concentration of 0.2 mg/mL (the second four samples), or the mixture of these two (the last four samples) was pre-incubated with the vehicle or different concentrations (0.5, 2 and 10 μM) of myricetin at room temperature for 20 min followed by an incubation with Alexa FluorTM 488 C5-maleimide at a final concentration of 1 μM for another 20 min. Samples were resolved on 12.5% acrylamide SDS-PAGE gel and visualized by in-gel fluorescence scanning. Three independent experiments were performed.

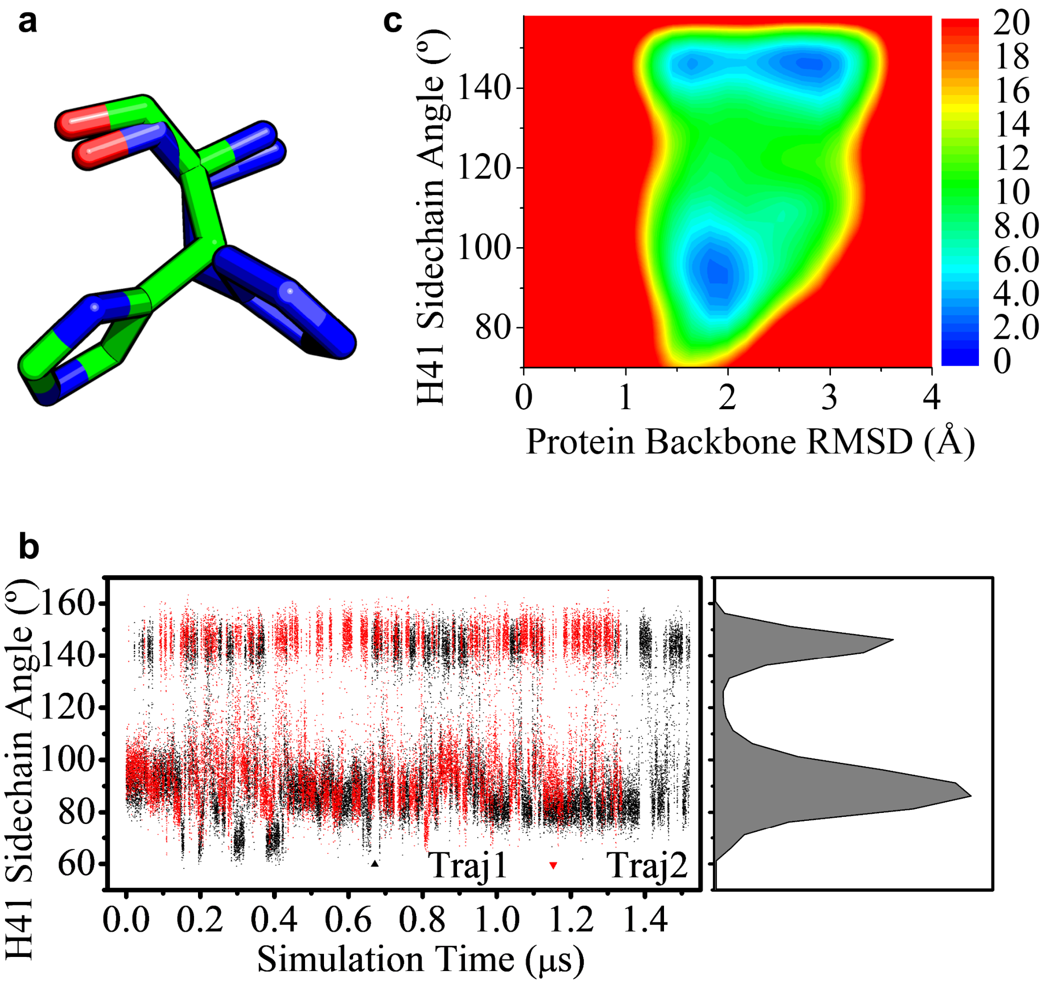

Supplementary Fig. 6 a, His41 side chain angles in the two identified configurations. **b**, Time series of the His41 side chain angle in GaMD simulation trajectories of apo SARS-CoV-2 3CL^{pro} dimer in aqueous solution. **c**, Two-dimensional free energy landscape as a function of protein backbone RMSD (root-mean-square deviation) and His41 side chain angle. The contours are spaced at intervals of 0.5 kcal/mol.

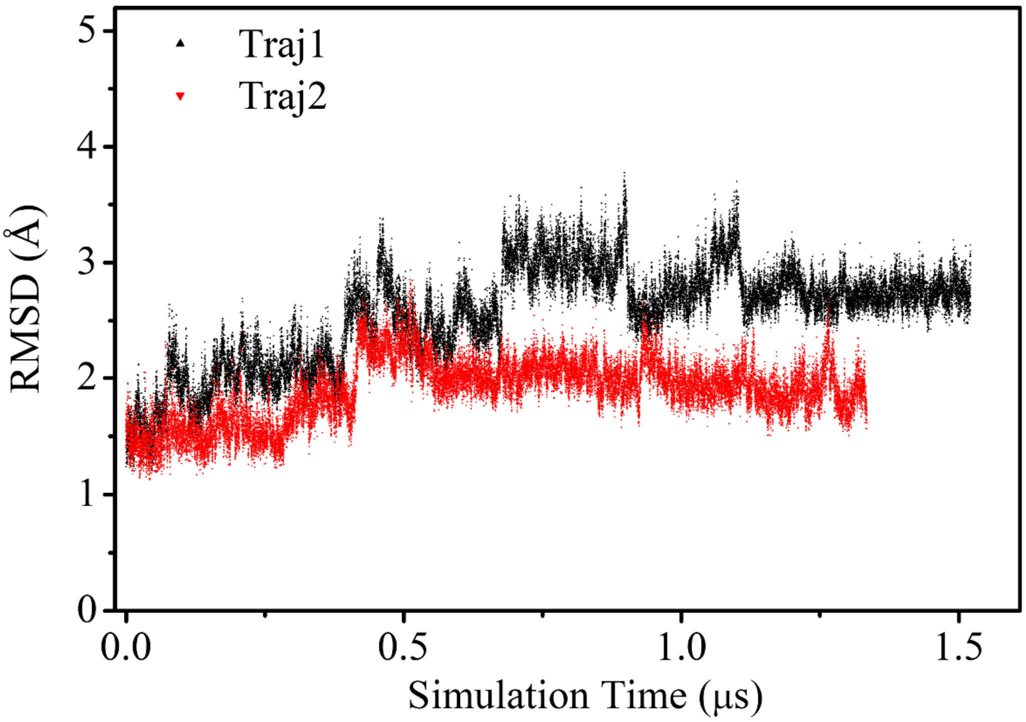

Supplementary Fig. 7 Time series of protein backbone RMSD in individual GaMD simulation trajectories of apo SARS-CoV-2 3CL^{pro} dimer in aqueous solution. The RMSD is calculated with respect to the crystal structure of 6M2Q**.**

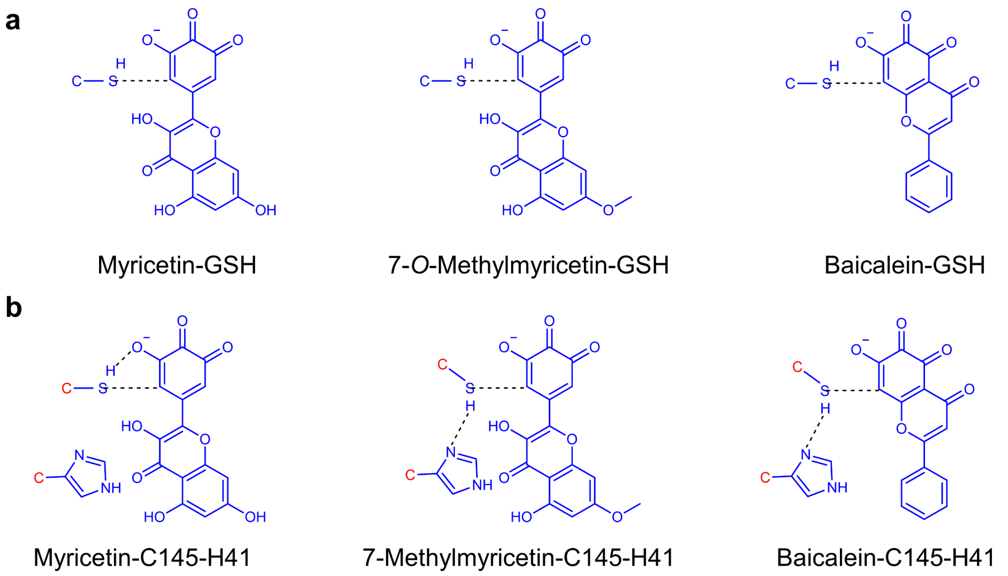

Supplementary Fig. 8 Models for the systems of thiol adducting with myricetin, 7-*O*-methylmyricetin and baicalein. a, models of myricetin, 7-*O*-methylmyricetin, and baicalein *o*-quinones reacting with GSH in aqueous solution and **b**, models of myricetin, 7-*O*-methylmyricetin, and baicalein *o*-quinones reacting with cysteine and histidine as in the SARS-CoV-2 3CL^{pro}. The boundary atoms (red colored) are fixed at the position as they are in the protein environment during the quantum chemistry calculation.

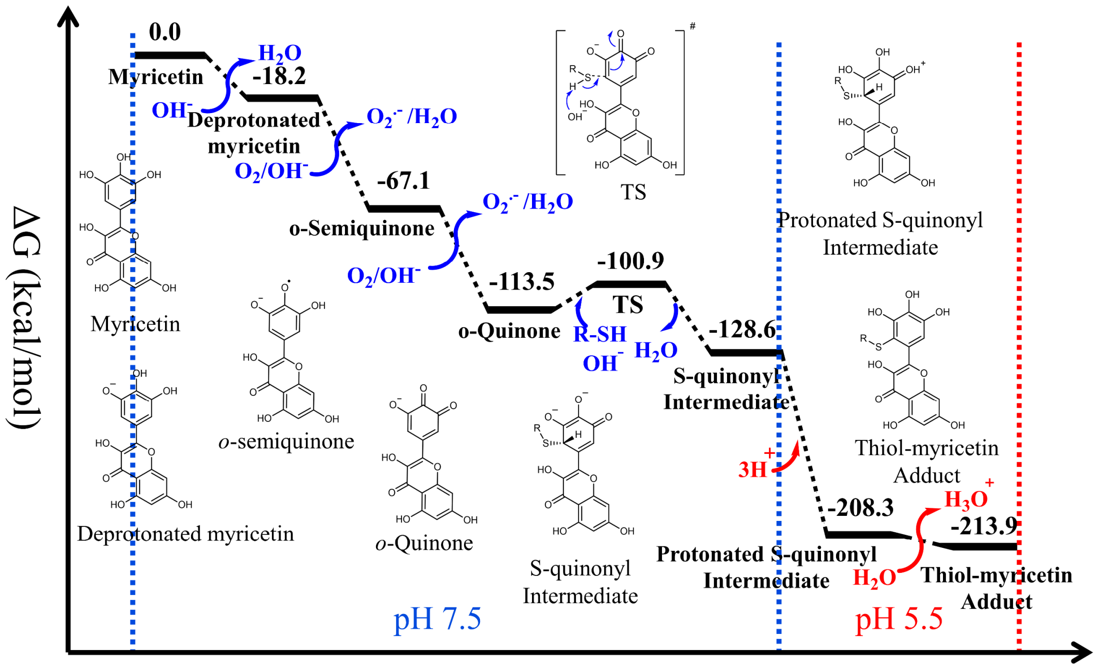

Supplementary Fig. 9 The free energy change along the reaction of myricetin and GSH with the pH values indicated in the corresponding experimentally measured reaction process.

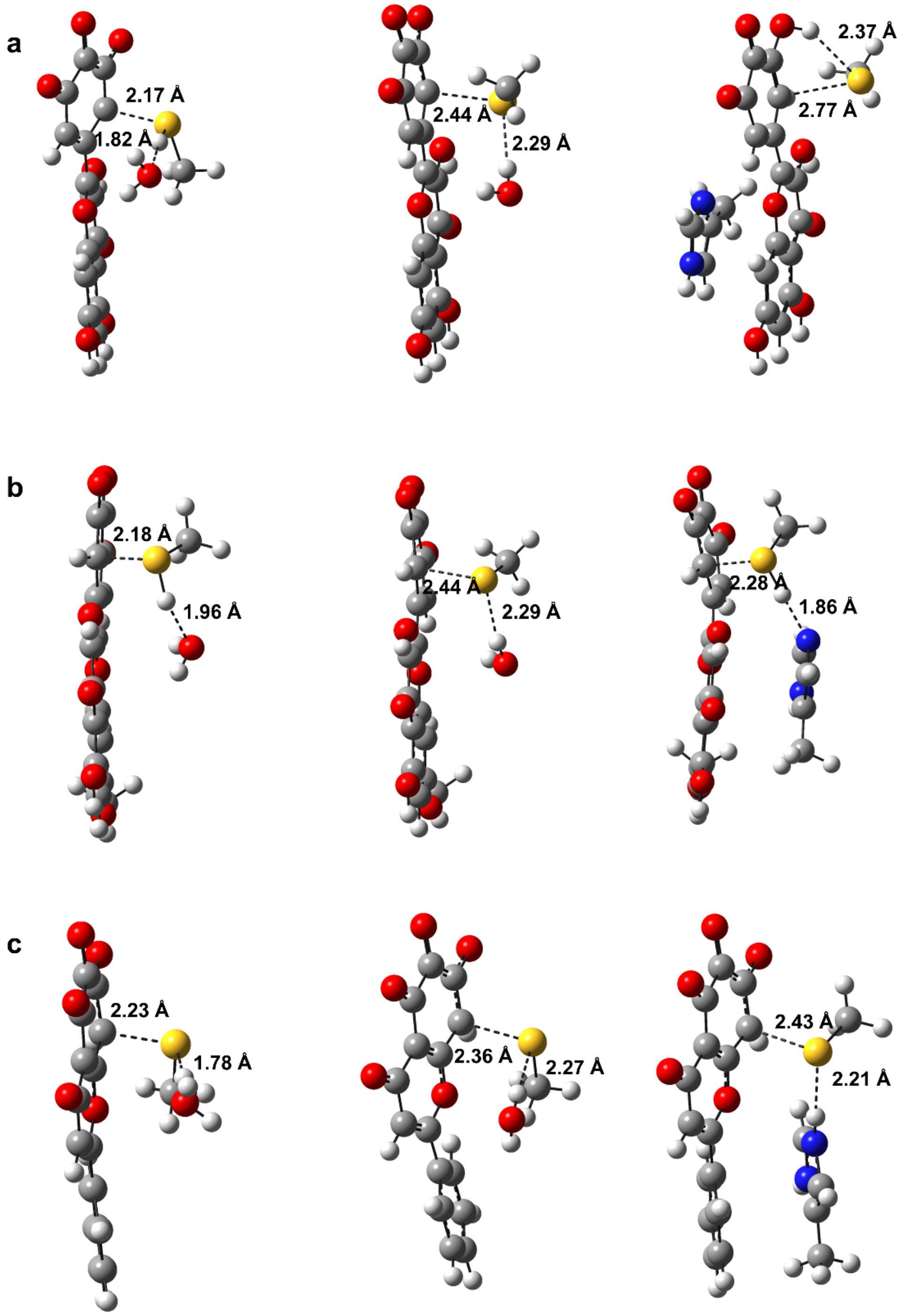

Supplementary Fig. 10 The optimized transition state (TS) structures for the adduction reaction of thiol with myricetin (**a**), 7-*O*-methylmyricetin (**b**) and baicalein *o*-quinones (**c**). From the left to right panel are the TS structures in the presence of water, OH^- and histidine. The structures are represented as ball and stick. The distances shown in black dash lines suggest the existence of intermolecular interactions. Values are given in angstrom.

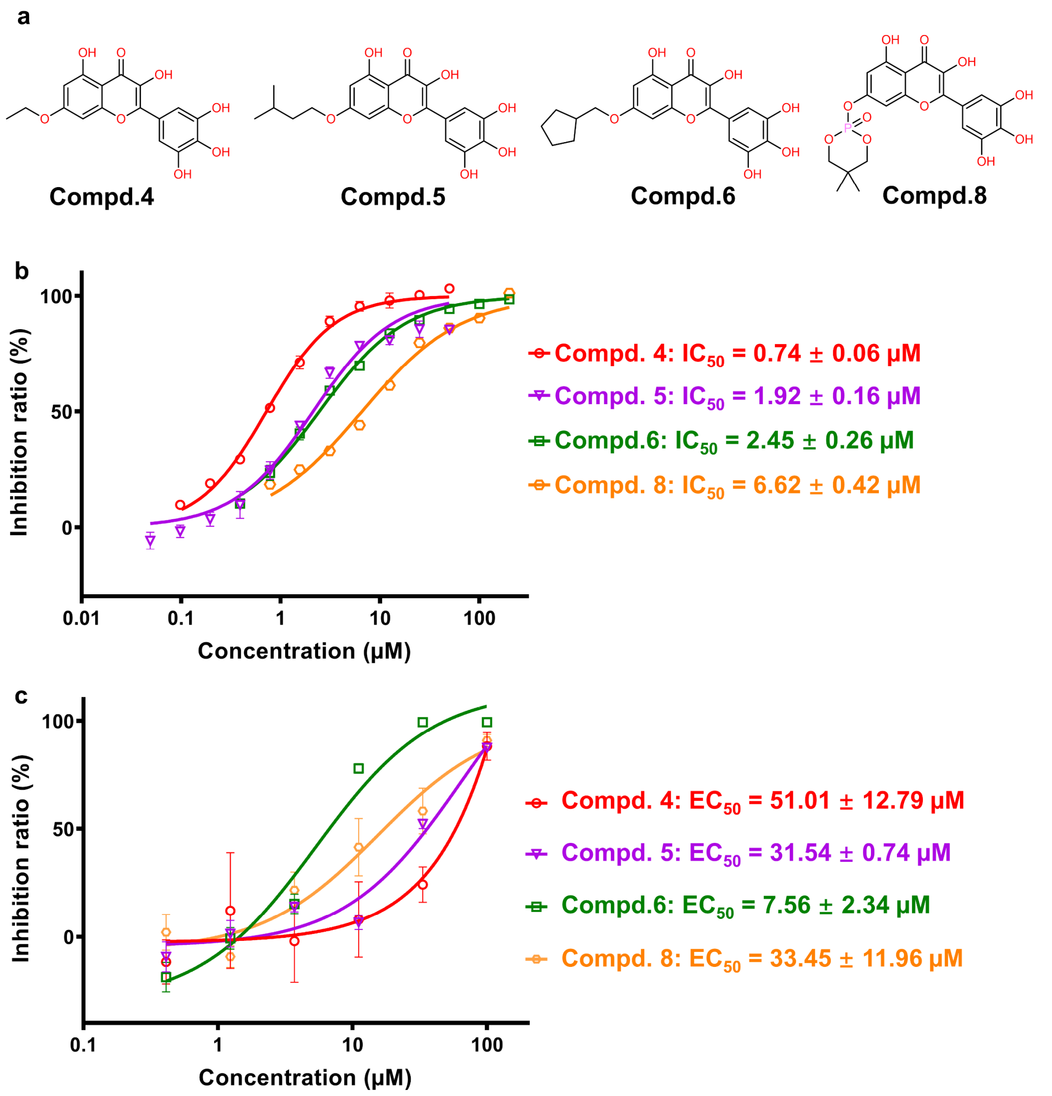

Supplementary Fig. 11 Inhibition of the enzymatic activity of SARS-CoV-2 3CL^{pro} and the replication of SARS-CoV-2 in cells by myricetin derivatives. a, Chemical structures of compounds **4**, **5**, **6**, and **8**. **b**, Representative inhibition curves for compounds **4** (red), **5** (purple), **6** (green), and **8** (orange) against the SARS-CoV-2 3CL^{pro}. **c**, Inhibition curves of compounds **4** (red), **5** (purple), **6** (green), and **8** (orange) against the replication of SARS-CoV-2 in Vero E6 cells. Error bars in Supplementary Fig. 11b,c represent mean ± SD of three independent experiments

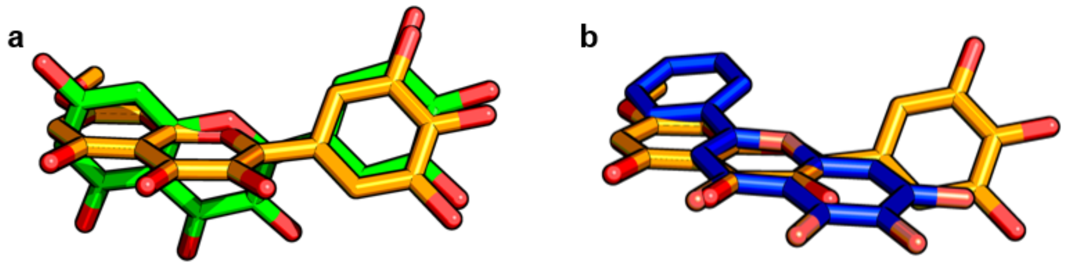

Supplementary Fig. 12 A superimposition of the binding poses of compound **3** (orange sticks) with that of myricetin (green sticks) (**a**) and baicalein (blue sticks) (**b**), respectively.

III. Supplementary Tables

Supplementary Table 1. Inhibition of 19 flavonoids on the SARS-CoV-2 3CL^{pro}

Compound	Structure	Inhibition (%) @10 μM
Biochanin A	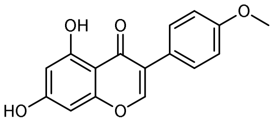	5.0
Daidzein	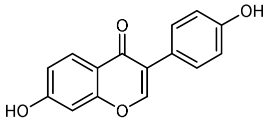	13.9
Genistin	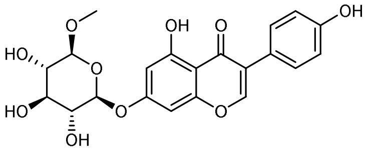	25.5
Formononetin	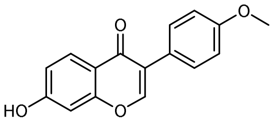	16.0
Hyperoside	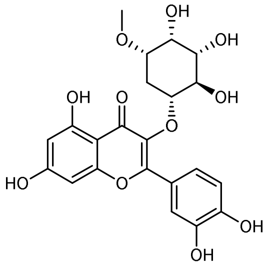	5.2
Hesperetin	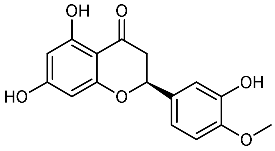	13.8
(+)-Catechin	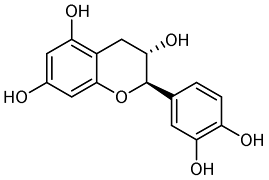	14.0
Taxifolin	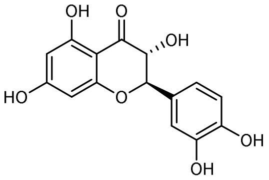	28.0
Kaempferide	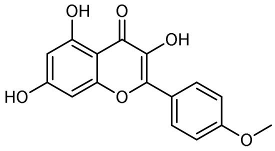	8.1

Supplementary Table 1 (continued). Inhibition of 19 flavonoids on the SARS-CoV-2 $3CL^{pro}$

Compound	Structure	Inhibition (%) @10 μM
Apigenin	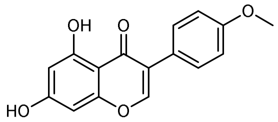	-1.0
Quercetin	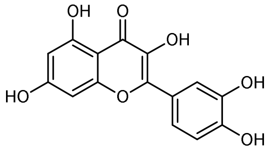	41.3
Eriodictyol	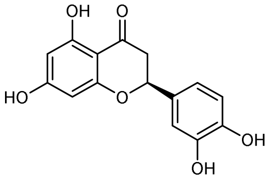	34.5
Luteolin	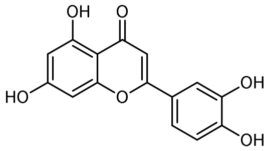	-4.1
Isorhamnetin	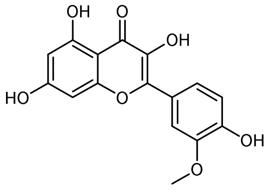	-2.6
Diosmetin	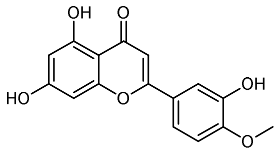	11.3
Sophoricoside	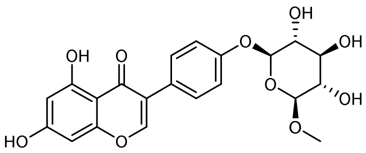	10.0
Genistein	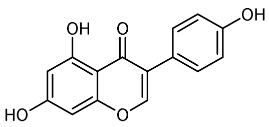	15.0
Myricetin	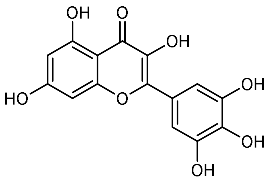	97.6

Supplementary Table 1 (continued). Inhibition of 19 flavonoids on the SARS-CoV-2 $3CL^{pro}$

Compound	Structure	Inhibition (%) @10 μM
Dihydromyricetin	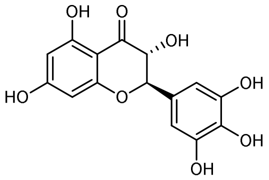	93.8

Supplementary Table 2. Inhibition of myricetin and its derivatives on the SARS-CoV-2 3CL^{pro} and the replication of SARS-CoV-2 in cells.

**Compd.**	**Structure**	**cLogP**	**IC$_{50}$ (μM)**	**EC$_{50}$ (μM)**
Myricetin (**1**)	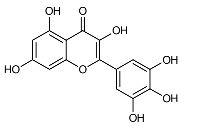	0.84	0.63 ± 0.01	8.00 ± 2.05
Dihydromyricetin (**2**)	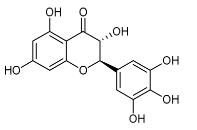	0.10	1.14 ± 0.03	13.56 ± 2.50
(**3**)	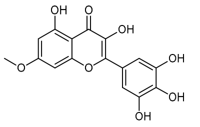	1.48	0.30 ± 0.00	12.59 ± 4.41
(**4**)	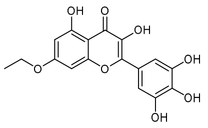	2.01	0.74 ± 0.06	51.01 ± 12.79
(**5**)	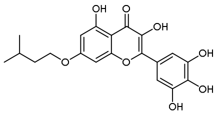	3.47	1.92 ± 0.16	31.54 ± 0.74
(**6**)	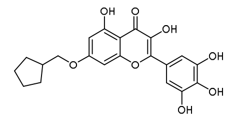	3.57	2.45 ± 0.26	7.56 ± 2.34
(**7**)	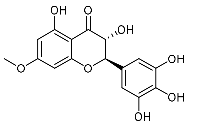	0.63	0.26 ± 0.02	11.50 ± 4.57
(**8**)	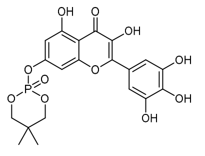	2.26	6.62 ± 0.42	33.45 ± 11.96
(**9**)	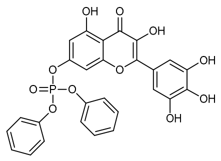	3.89	3.13 ± 0.37	3.15 ± 0.84
(**10**)	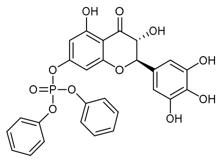	3.07	1.84 ± 0.22	9.03 ± 1.36

Supplementary Table 3. Crystallography data collection and refinement statistics.

	$3CL^{pro}$-myricetin	$3CL^{pro}$-**3**	$3CL^{pro}$-**7**
**PDB ID**	7DPP	7DPU	7DPV
Space Group	*C 1 2 1*	*P 1 2(1) 1*	*P 1 2(1) 1*
Cell Dimension: a (Å)	97.16	44.26	88.65
b (Å)	83.25	53.80	68.05
c (Å)	51.69	115.06	102.39
Wavelength (Å)	0.979	0.979	0.979
Reflections (unique)	21684	52603	50745
Resolution Range (Å)	46.75-2.10	48.57-1.75	44.34-2.35
Highest-Resolution Shell (Å)	2.19-2.10	1.81-1.75	2.43-2.35
Redundancy	6.6 (4.8)	6.8 (6.7)	4.1 (3.1)
I/σ (I)	16.7 (1.4)	20.2 (2.0)	7.9 (1.6)
Highest-Resolution Shell $CC_{1/2}$	0.710	0.760	0.681
Completeness (%)	99.7 (97.2)	97.7 (95.3)	99.2 (92.1)
Rwork/Rfree	0.185/0.220	0.165/0.199	0.202/0.234
**RMS Values**			
Bond length (Å)	0.004	0.004	0.002
Bond angle (°)	0.583	0.708	0.455
**Numbers of Non-hydrogen Atoms**			
Protein	2230	2661	8605
Inhibitor	23	48	96
Water Oxygen	67	410	297
Others	0	6	0
Clashscore	1.37	1.41	1.79
MolProbity Score	0.87	0.87	0.98
**B-factor ($Å^2$)**			
Protein	46.93	23.30	41.07
Inhibitor	62.02	23.36	65.00
Water Oxygen	45.00	32.15	34.22
**Ramachandran plot**			
Favored (%)	98.28	98.50	97.81
Allowed (%)	1.72	1.50	2.19
Outliers (%)	0	0	0

Supplementary Table 4. PK Profiles of compound **7** and myricetin in Mice.

Compound	**7**		Myricetin	
Administration	p.o. (30 mg/kg)	i.v. (10 mg/kg)	p.o. (30 mg/kg)	i.p. (10 mg/kg)
$T_{1/2}$ (h)	1.74 ± 0.83	0.58 ± 0.63	0.44	2.10 ± 0.02
T_{max} (h)	0.25 ± 0.00	\	0.50	\
C_{max} (ng/mL)	724 ± 223	\	8.59	1799 ± 346
AUC_{last} (ng•h/mL)	510 ± 99	940 ± 374	6.07	1087 ± 103
$CL_{\_obs}$ (mL/min/kg)	\	230 ± 193	\	\
MRT (h)	1.89 ± 0.58	0.69 ± 0.86	0.84	0.09
$Vss_{\_obs}$ (mL/kg)	\	6196 ± 5909	\	\
F (%)	18.1	\	\	\

Supplementary Table 5. A list of primers used.

Names	Sequences
RBD-qF1	5’-CAATGGTTTAACAGGCACAGG-3’
RBD-qR1	5’-CTCAAGTGTCTGTGGATCACG-3’

Supplementary Table 6. Summary of M06-2*X* calculated energies for the optimized geometries in the auto-oxidization reaction of myricetin, 7-*O*-methylmyricetin and baicalein.

Geometry	Single-point energy (a.u.)	Thermal correction of Gibbs free energy (a. u.)	Gibbs free energy (a. u.)	IF (cm^{-1})
Myricetin	-1179.35586371	0.185925	-1179.169939	
Deprotonated myricetin	-1178.89018465	0.174662	-1178.715523	
Myricetin *o*-semiquinone	-1178.26937440	0.162352	-1178.107023	
Myricetin *o*-quinone	-1177.64516718	0.149880	-1177.495287	
7-*O*-Methylmyricetin	-1218.63927273	0.213172	-1218.426101	
Deprotonated 7-*O*-methylmyricetin	-1218.17353437	0.200986	-1217.972548	
7-*O*-Methylmyricetin *o*-semiquinone	-1217.55270625	0.189053	-1217.363653	
7-*O*-Methylmyricetin *o*-quinone	-1216.92862767	0.176501	-1216.752126	
Baicalein	-953.671185218	0.177999	-953.493186	
Deprotonated baicalein	-953.204195756	0.164349	-953.039847	
Baicalein *o*-semiquinone	-952.584058891	0.152702	-952.431357	
Baicalein *o*-quinone	-951.968816728	0.141132	-951.827685	

Single-point energies, thermal corrections of Gibbs free energies, and Gibbs free energies were calculated at the M06-2*X*/6-311++G(d,p) level. Gibbs free energies are equal to the addition of single-point energies and thermal corrections of Gibbs free energies. Imaginary frequency (IF) is available only for transition states. The abbreviation ‘a.u.’ means ‘atomic units’.

Supplementary Table 7. Summary of M06-2*X* calculated energies for optimized geometries in the adduction reaction of GSH and myricetin, 7-*O*-methylmyricetin, or baicalein in neutral solution.

Geometry	Single-point energy (a.u.)	Thermal correction of Gibbs free energy (a. u.)	Gibbs free energy (a. u.)	IF (cm^{-1})
Me-SH	-438.677298495	0.022338	-438.654961	
Myricetin				
Myricetin *o*-quinone	-1177.64516718	0.149880	-1177.495287	
TS	-1692.71841166	0.216858	-1692.501554	-592.13
S-quinonyl intermediate	-1615.87680871	0.186417	-1615.690392	
Protonated S-quinonyl intermediate	-1617.24494291	0.224797	-1617.020146	
Thiol-myricetin adduct	-1616.83942072	0.211348	-1616.628073	
7-Methylmyricetin				
7-*O*-Methylmyricetin *o*-quinone	-1216.92862767	0.176501	-1216.752126	
TS	-1732.01025049	0.244739	-1731.765511	-533.12
S-quinonyl intermediate	-1655.16337633	0.213362	-1654.950015	
Baicalein				
Baicalein *o*-quinone	-951.968816728	0.141132	-951.827685	
TS	-1467.02704740	0.207683	-1466.819364	-628.14
S-quinonyl intermediate	-1390.18819885	0.176605	-1390.011594	

Supplementary Table 8. Summary of M06-2*X* calculated energies for optimized geometries in the adduction reaction of GSH and myricetin, 7-*O*-methylmyricetin, or baicalein in alkaline solution.

Geometry	Single-point energy (a.u.)	Thermal correction of Gibbs free energy (a. u.)	Gibbs free energy (a. u.)	IF (cm^{-1})
Me-SH	-438.677298495	0.022338	-438.654961	
Myricetin				
Myricetin *o*-quinone	-1177.64516718	0.149880	-1177.495287	
TS	-1691.83592962	0.208722	-1691.627207	-374.10
S-quinonyl intermediate	-1615.87680871	0.186417	-1615.690392	
7-*O*-Methylmyricetin				
7-*O*-Methylmyricetin *o*-quinone	-1216.92862767	0.176501	-1216.752126	
TS	-1731.11321937	0.235630	-1730.877589	-374.11
S-quinonyl intermediate	-1655.16337633	0.213362	-1654.950015	
Baicalein				
Baicalein *o*-quinone	-951.968816728	0.141132	-951.827685	
TS	-1466.22636165	0.196211	-1466.030151	-473.05
S-quinonyl intermediate	-1390.18819885	0.176605	-1390.011594	

Supplementary Table 9. Summary of M06-2*X* calculated energies for the optimized geometries in the adduction reaction of Cys145 and myricetin, 7-*O*-methylmyricetin, or baicalein.

Geometry	Single-point energy (a.u.)	Thermal correction of Gibbs free energy (a. u.)	Gibbs free energy (a. u.)	IF (cm^{-1})
Me-SH	-438.677298495	0.022338	-438.654961	
Histidine	-265.511875525	0.071217	-265.440658	
Myricetin				
Myricetin *o*-quinone	-1177.64516718	0.149880	-1177.495287	
TS	-1881.82266013	0.253570	-1881.569090	-178.17
S-quinonyl intermediate	-1881.86457414	0.259283	-1881.605291	
Protonated S-quinonyl intermediate	-1882.76578102	0.285163	-1882.480618	
Thiol-myricetin adduct	-1882.36184974	0.269962	-1882.091888	
7-*O*-Methylmyricetin				
7-*O*-Methylmyricetin *o*-quinone	-1216.92862767	0.176501	-1216.752126	
TS	-1921.10175939	0.279944	-1920.821856	-487.73
S-quinonyl intermediate	-1921.15115180	0.304494	-1920.846658	
Protonated S-quinonyl intermediate	-1922.51212497	0.348303	-1922.163822	
Thiol-7-*O*-methylmyricetin adduct	-1922.10205340	0.307275	-1921.794778	
Baicalein				
Baicalein *o*-quinone	-951.968816728	0.141132	-951.827685	
TS	-1656.13601762	0.270032	-1655.865986	-453.18
S-quinonyl intermediate	-1656.17300795	0.283703	-1655.889305	

III. Spectra of myricetin derivatives

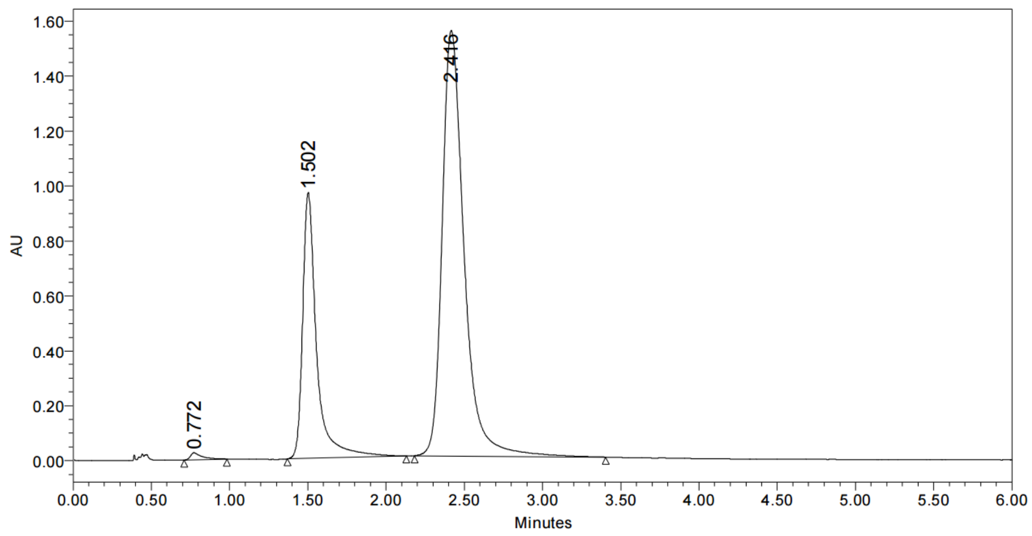

	RT	Area	% Area	Height
1	0.772	131944	0.62	25591
2	1.502	5996753	28.24	967850
3	2.416	15107895	71.14	1548215

The chiral HPLC chromatogram of commercial dihydromyricetin

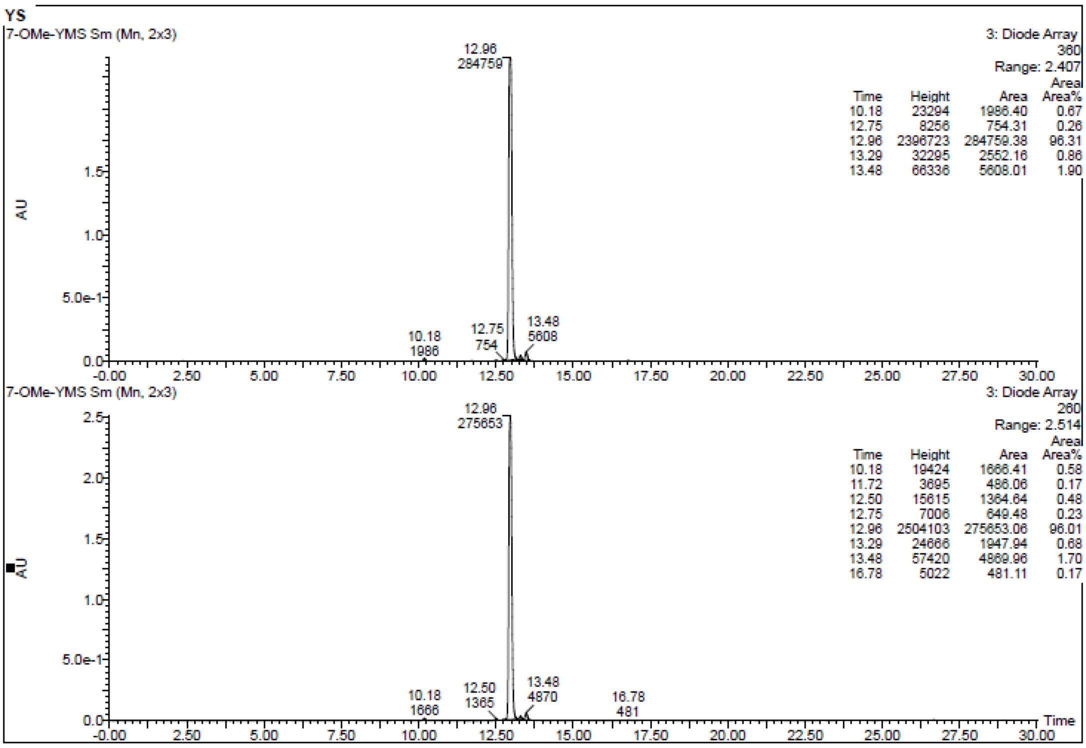

The purity of compound **3** at 360 nm and 260 nm

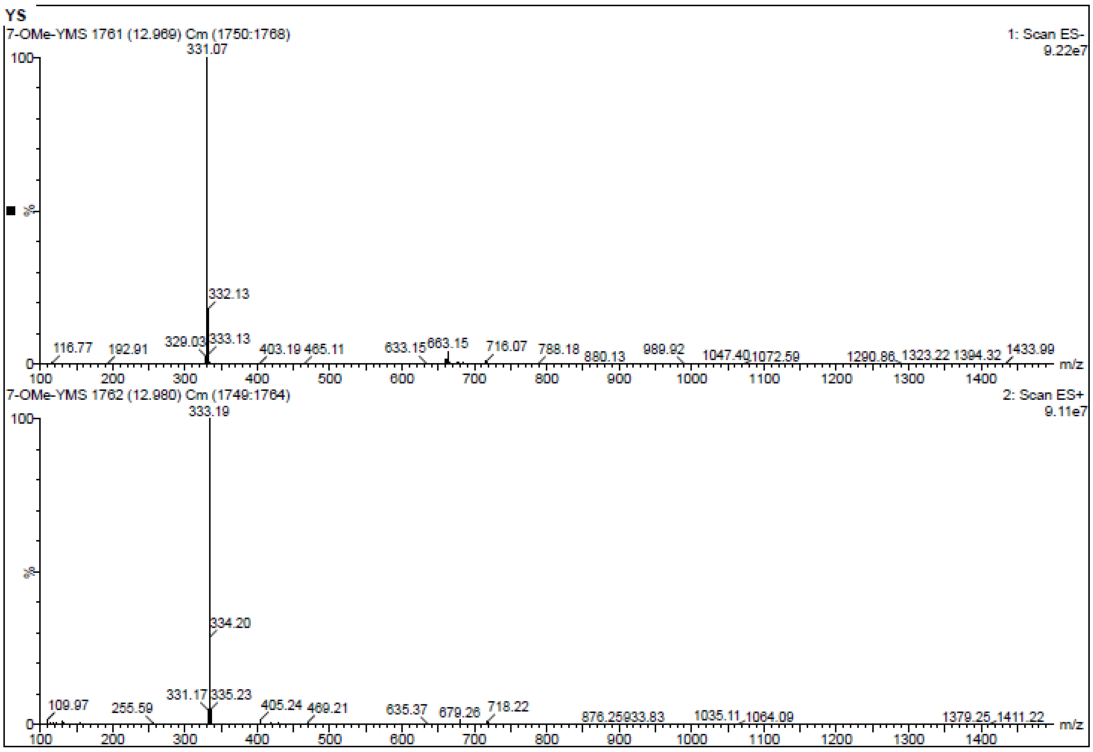

ESI-MS spectrum of compound **3**

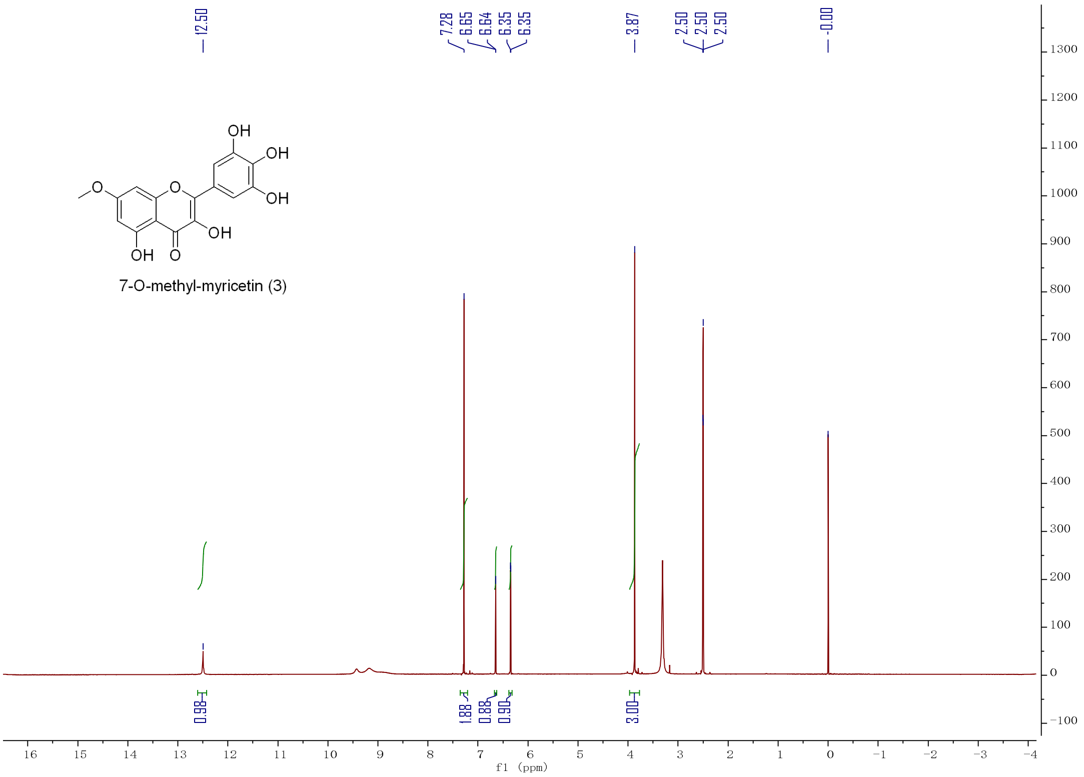

^{1}H NMR (DMSO-d_6, 500 MHz) spectrum of compound **3**

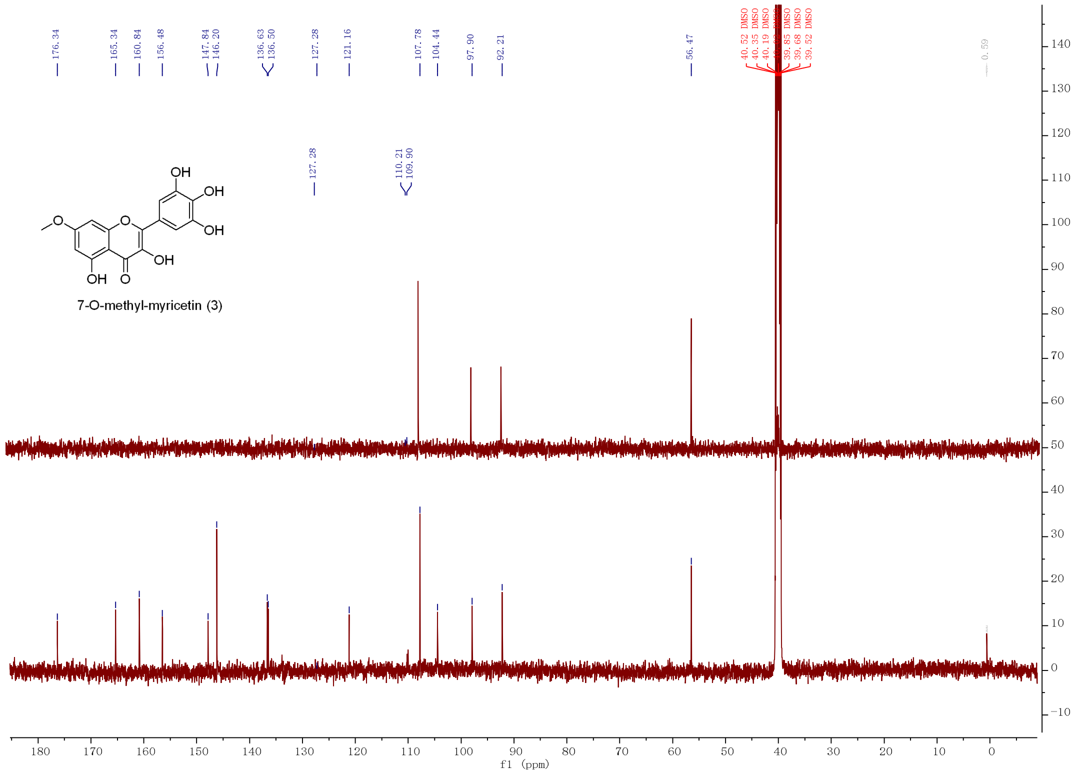

^{13}C NMR (DMSO-d_6, 125 MHz) and DEPT-135 spectra of compound **3**

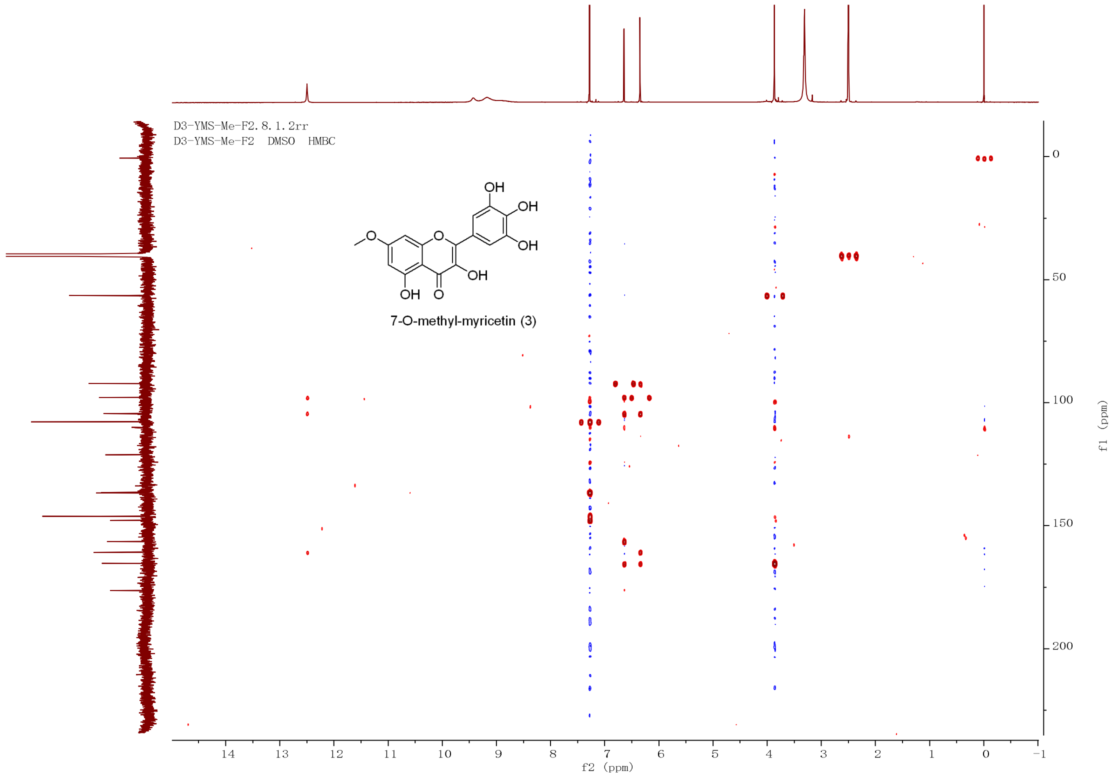

HMBC spectrum of compound **3**

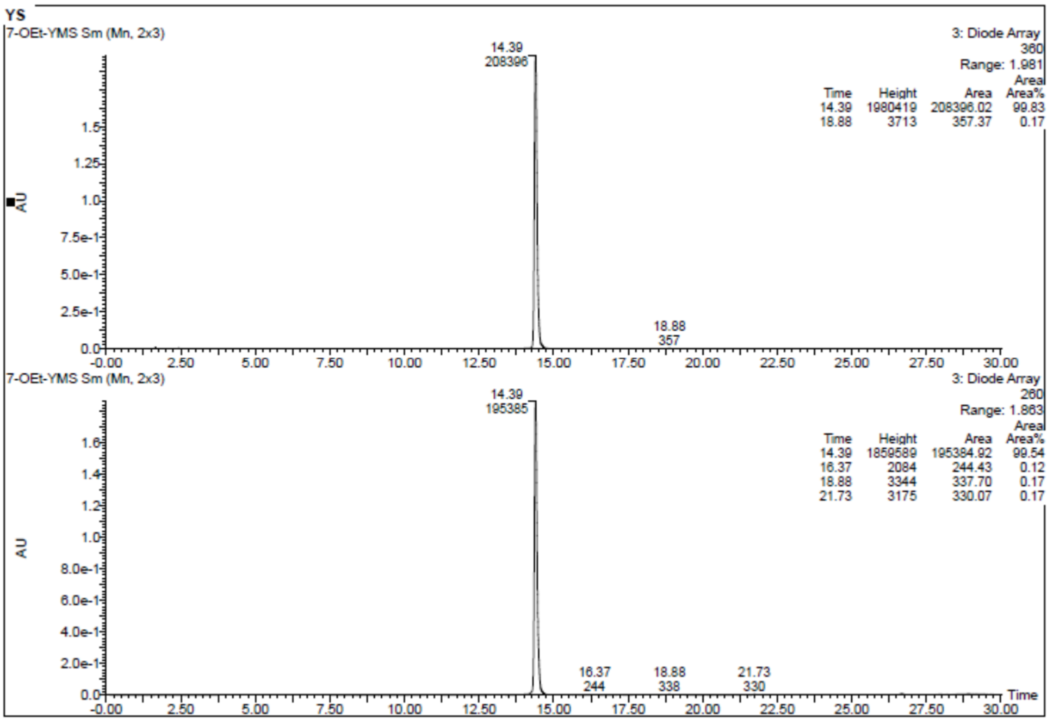

The purity of compound **4** at 360 nm and 260 nm

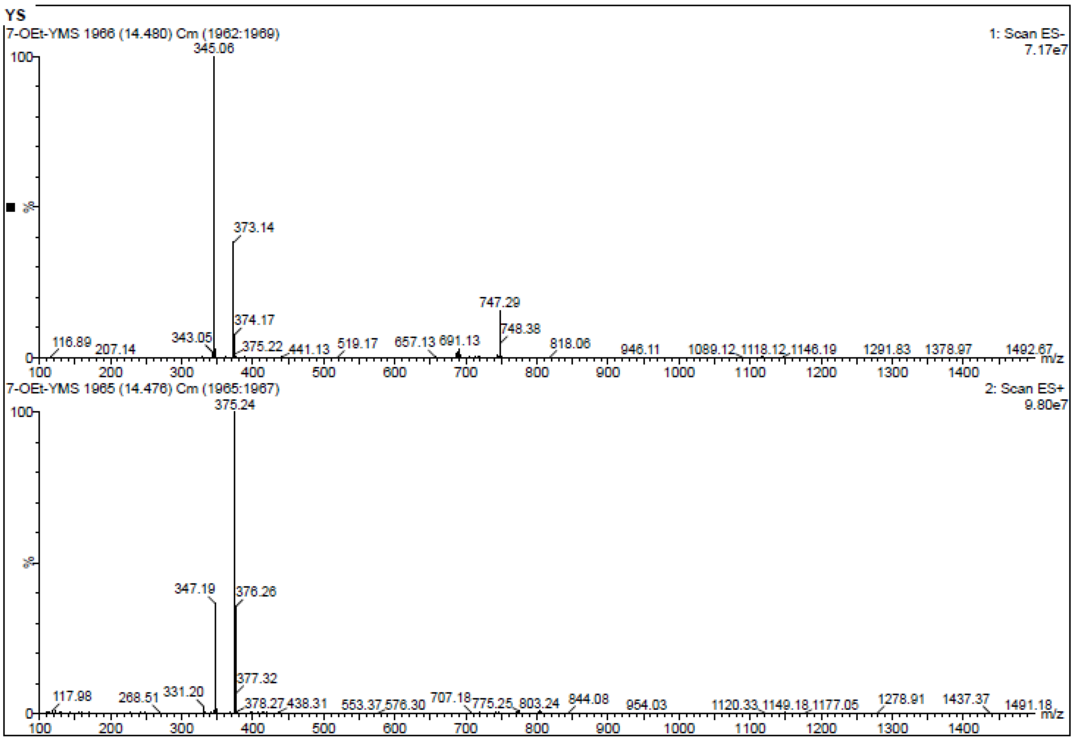

ESI-MS spectrum of compound **4**

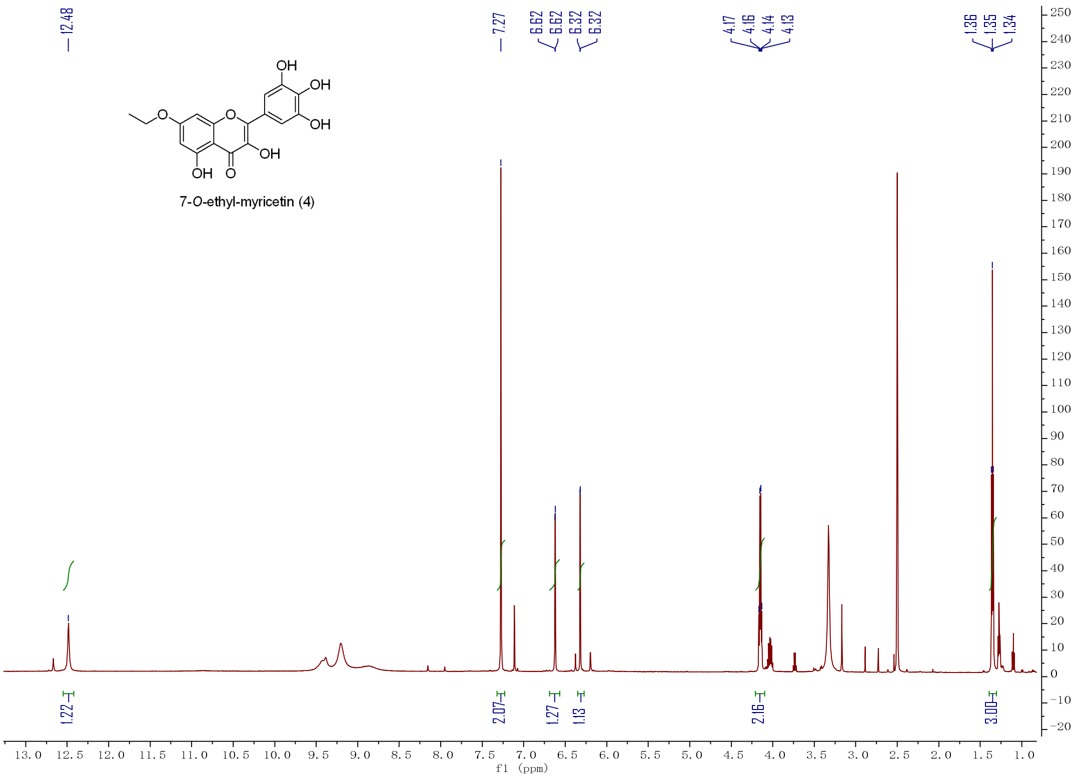

^{1}H NMR (DMSO-d_6, 500 MHz) spectrum of compound **4**

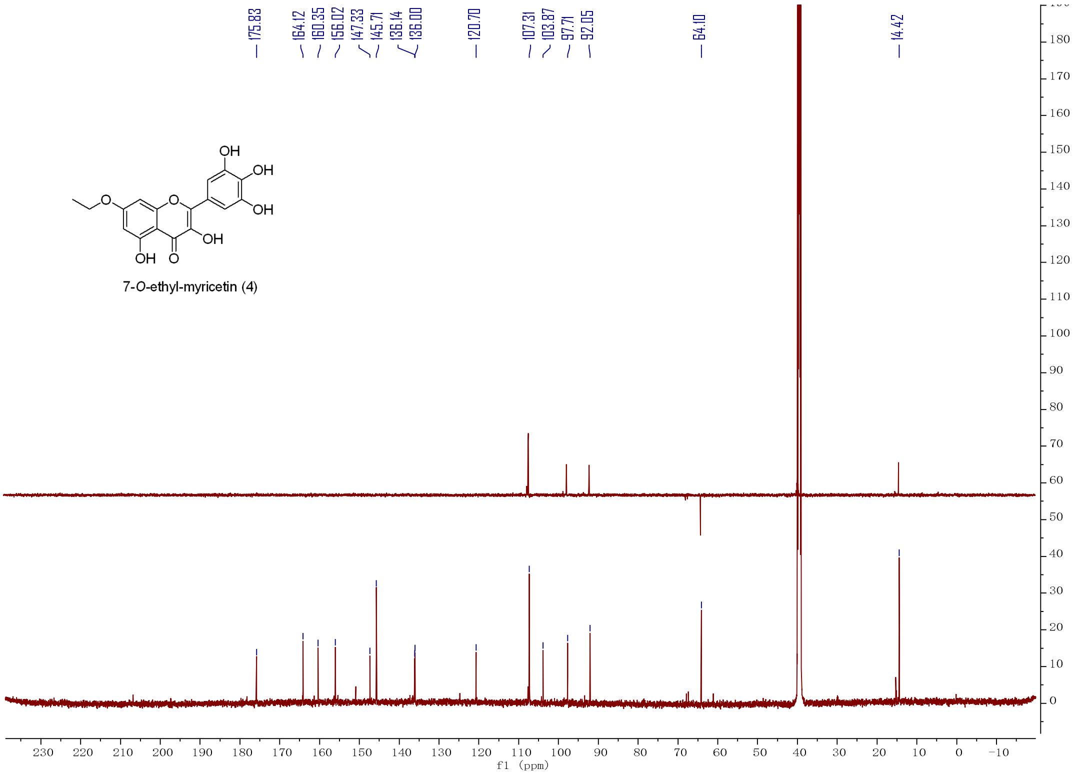

^{13}C NMR (DMSO-d_6, 125 MHz) and DEPT-135 spectra of compound **4**

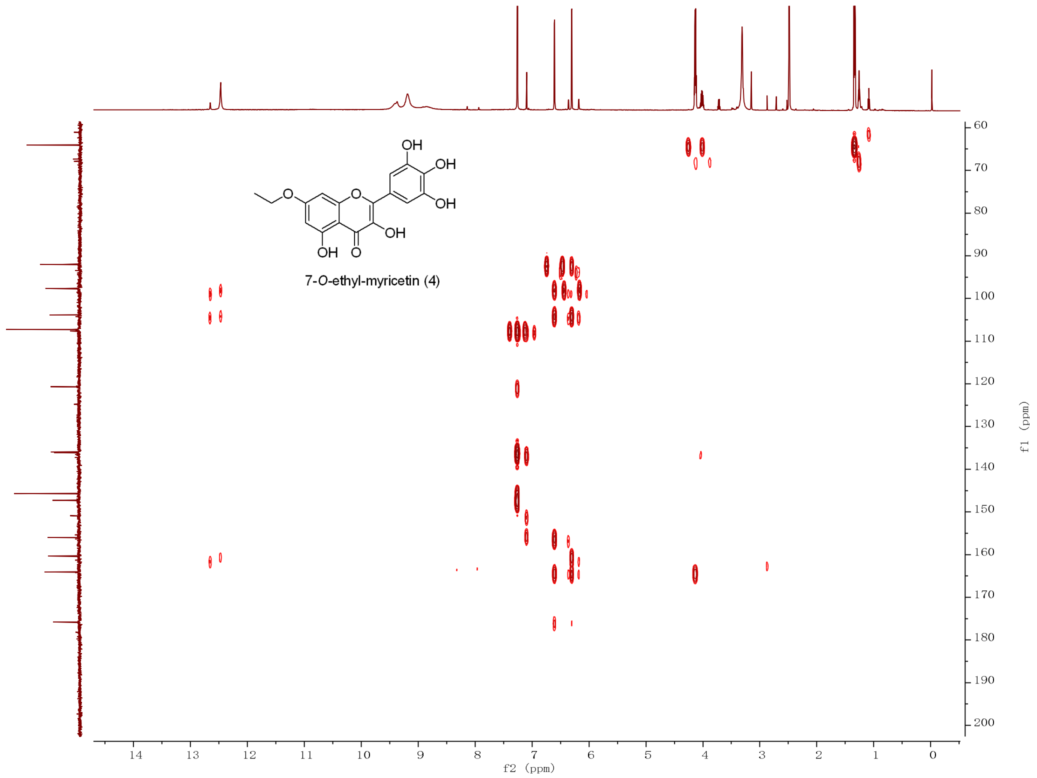

HMBC spectrum of compound **4**

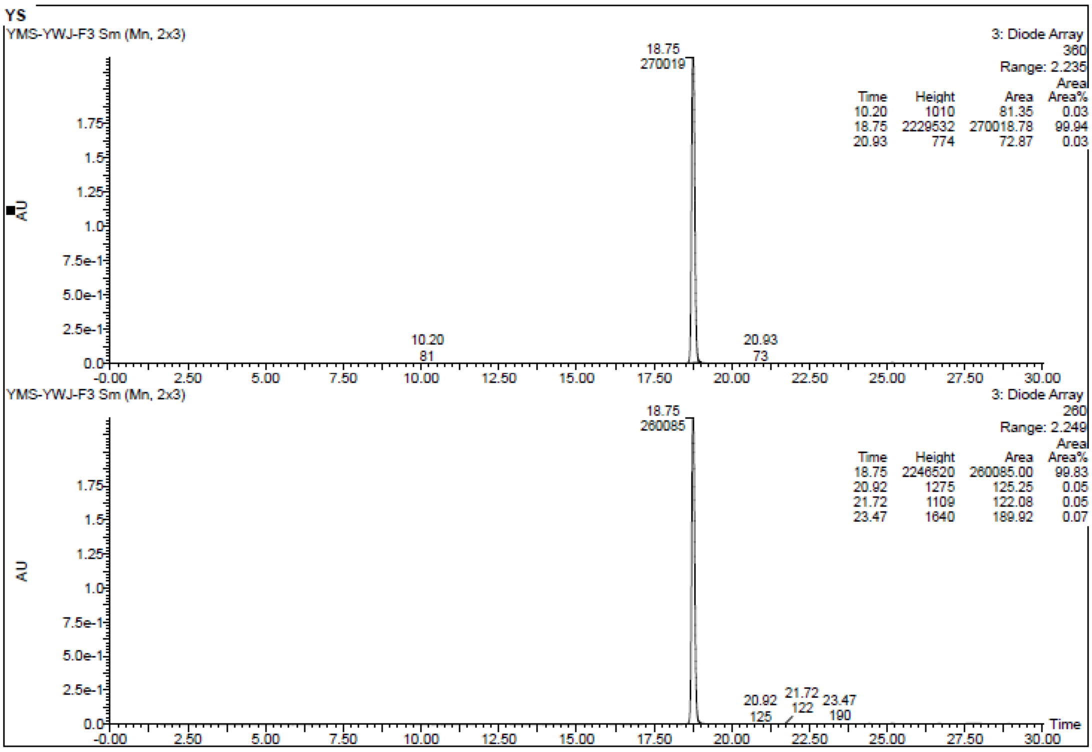

The purity of compound **5** at 360 nm and 260 nm

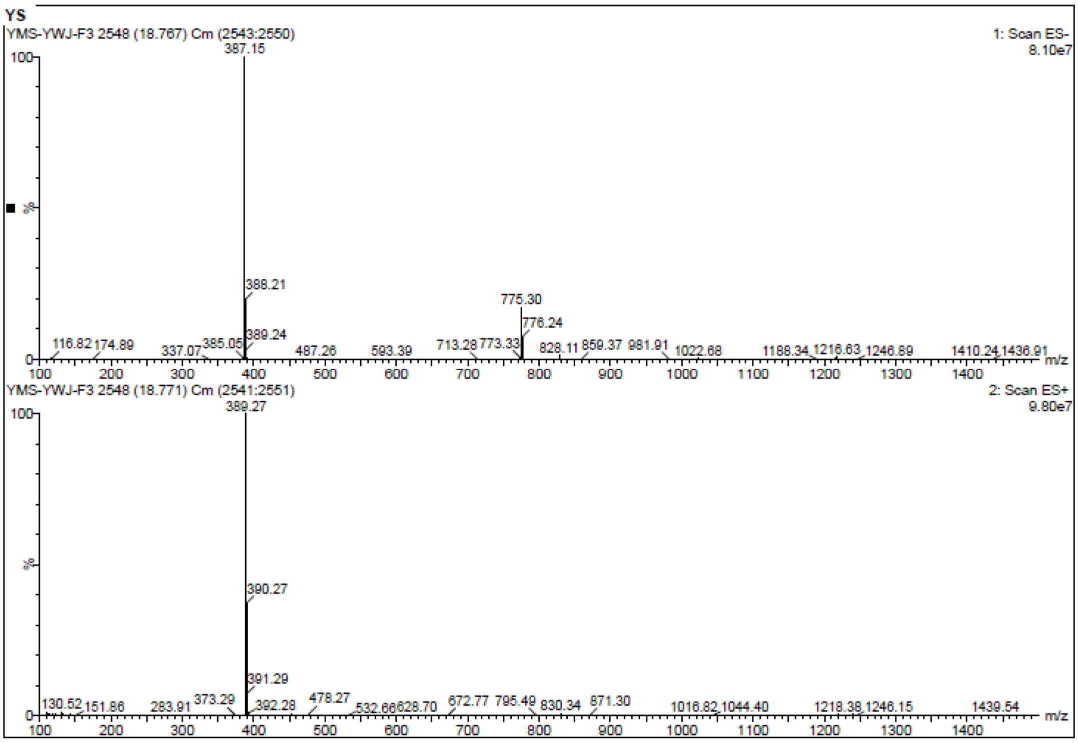

ESI-MS spectrum of compound **5**

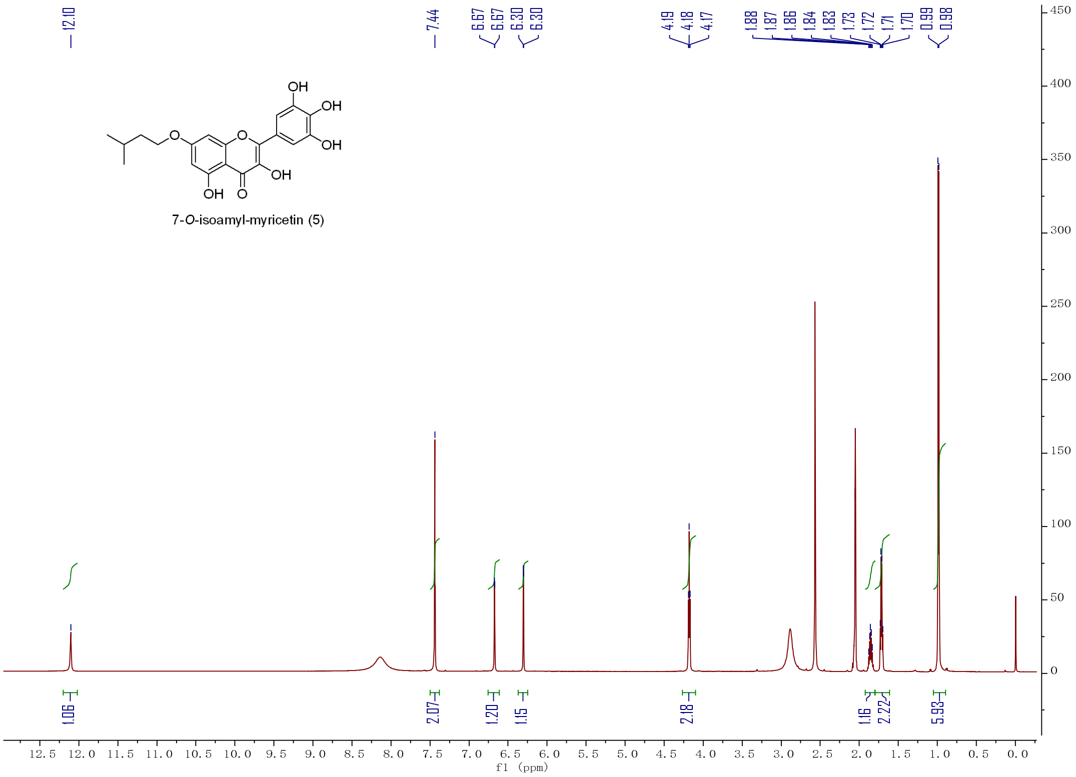

^{1}H NMR (Acetone-d_6, 500 MHz) spectrum of compound **5**

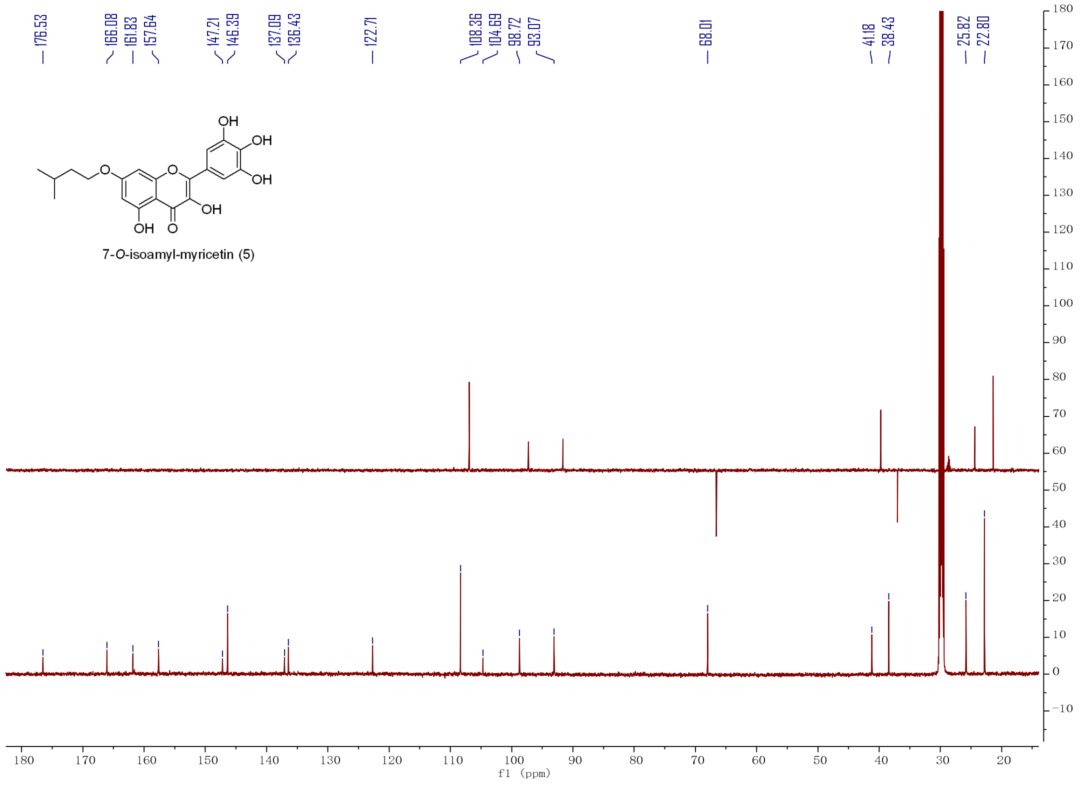

^{13}C NMR (Acetone-d_6, 125 MHz) and DEPT-135 spectra of compound **5**

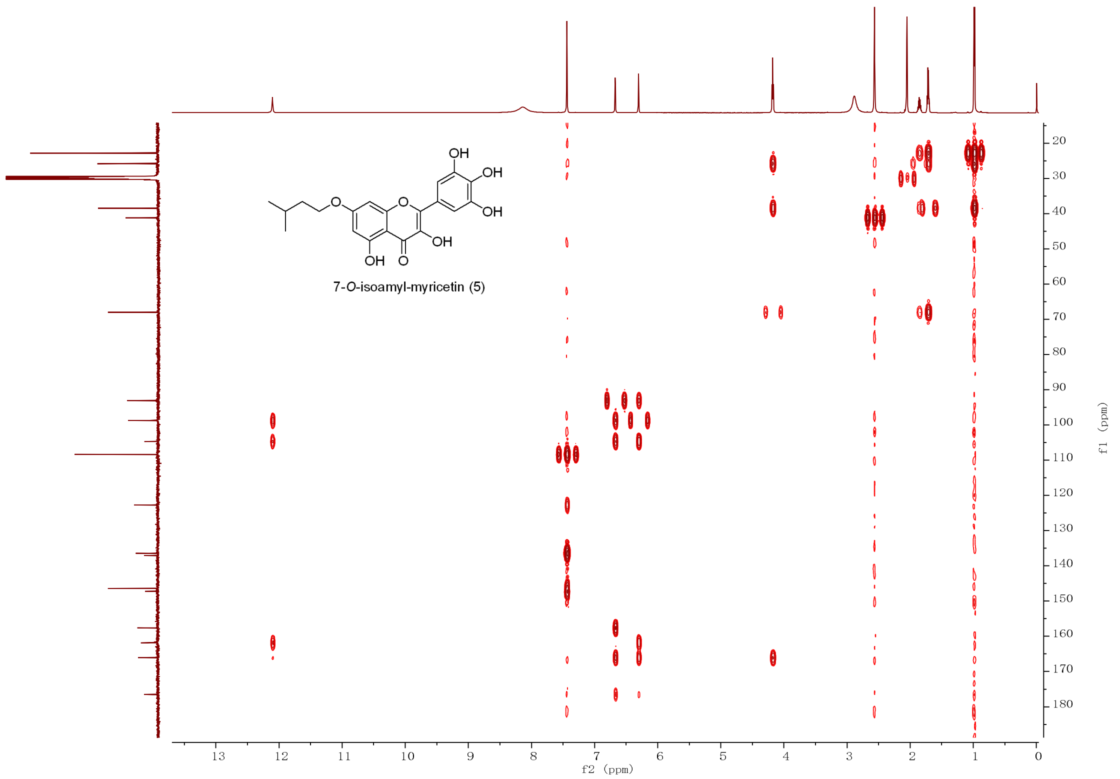

HMBC spectrum of compound **5**

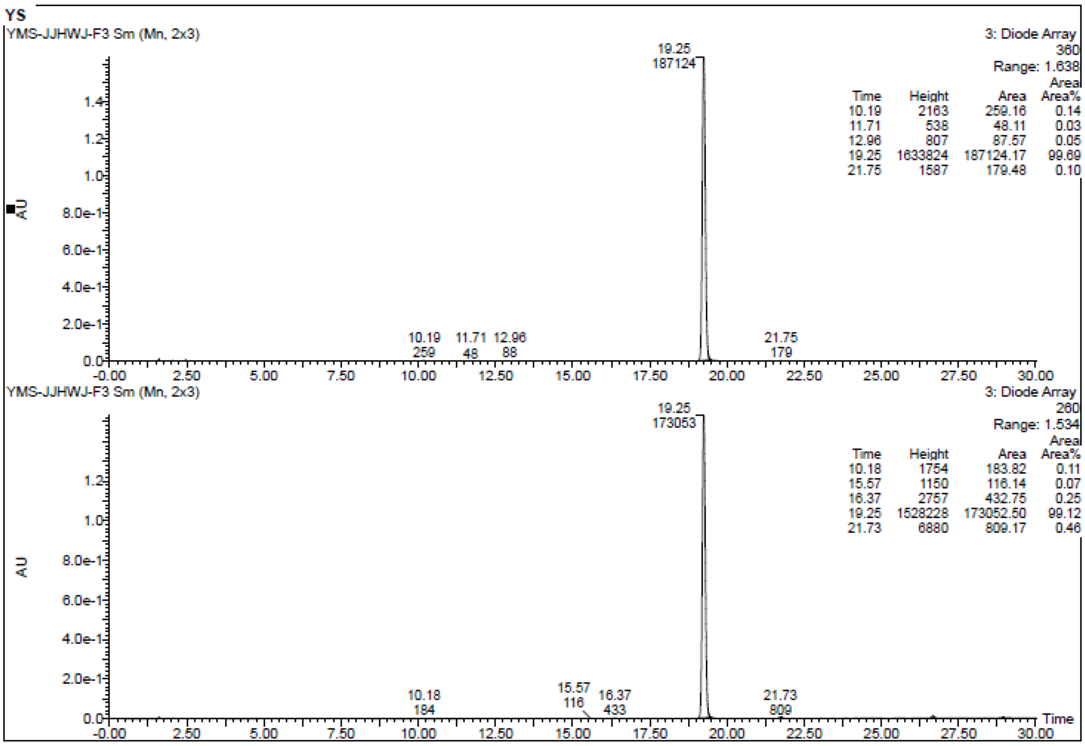

The purity of compound **6** at 360 nm and 260 nm

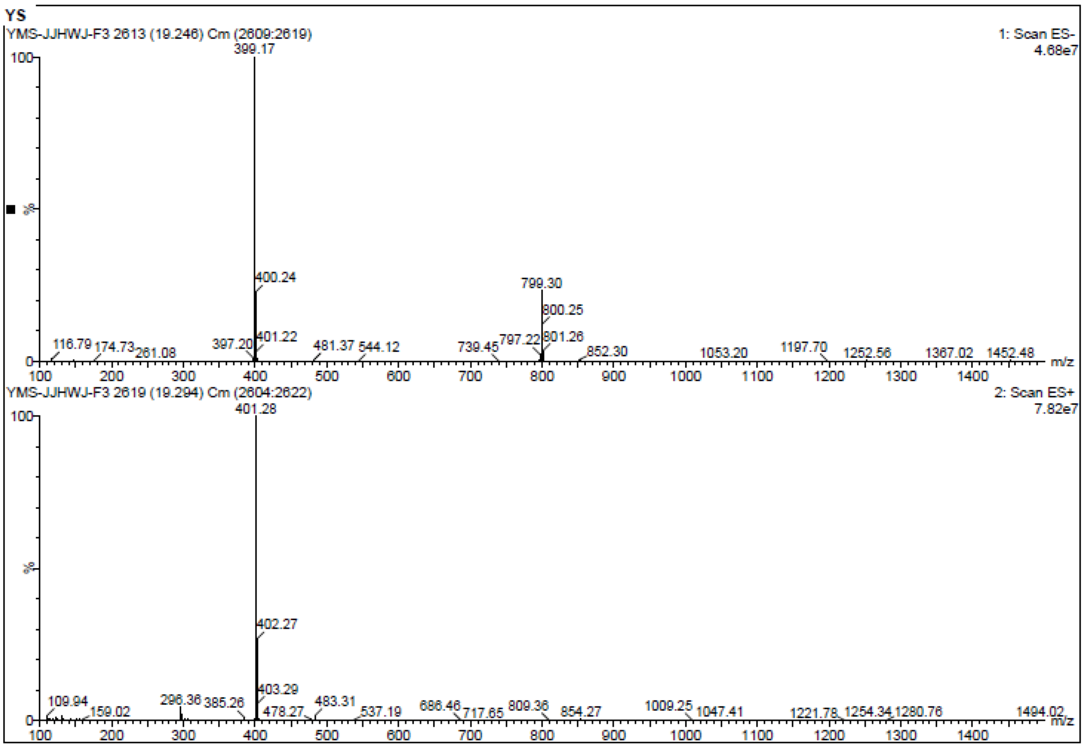

ESI-MS spectrum of compound **6**

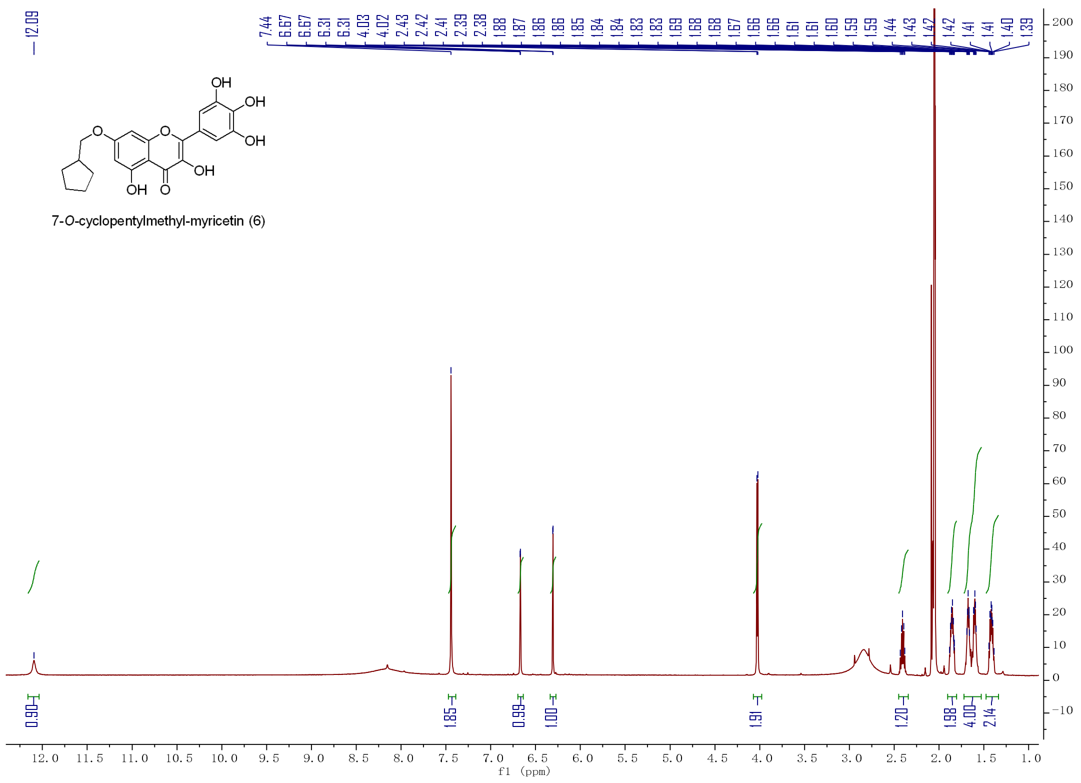

^{1}H NMR (Acetone-d_6, 600 MHz) spectrum of compound **6**

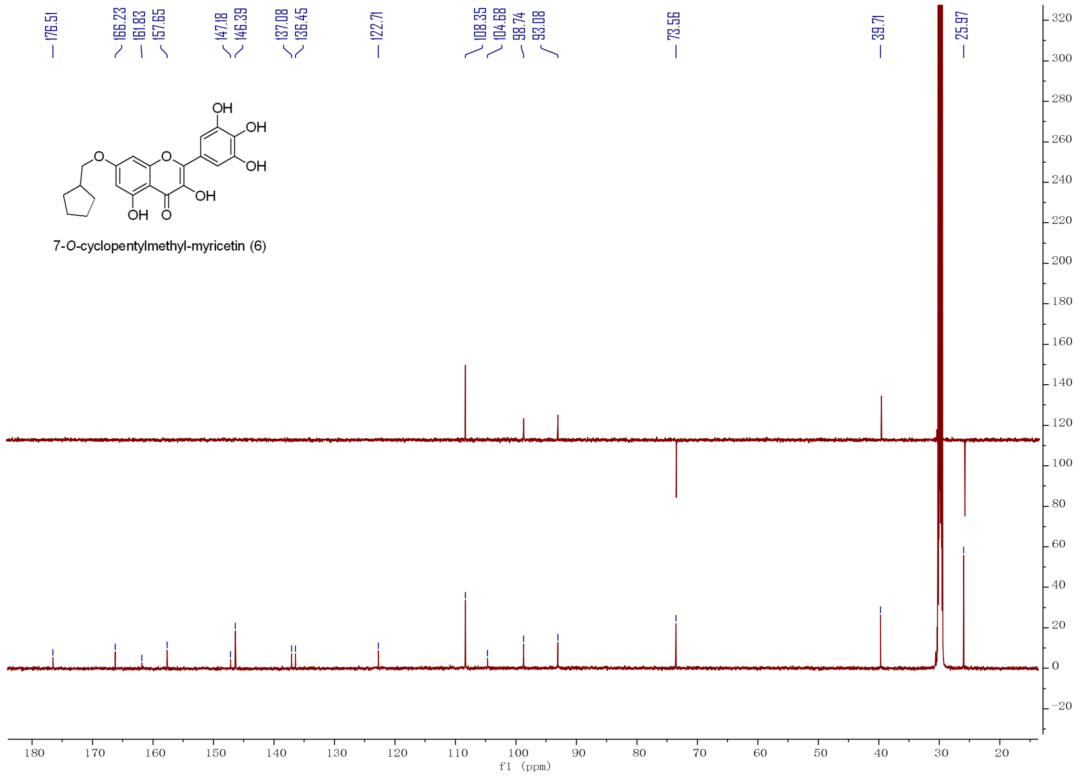

^{13}C NMR (Acetone-d_6, 150 MHz) and DEPT-135 spectra of compound **6**

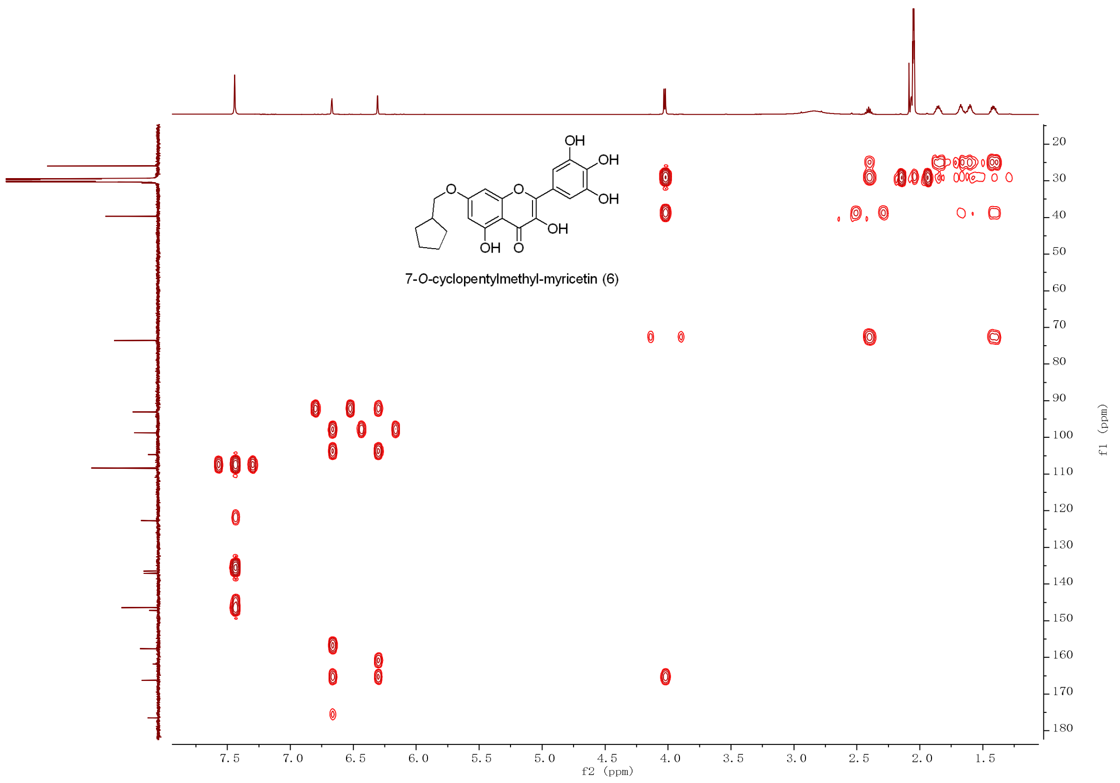

HMBC spectrum of compound **6**

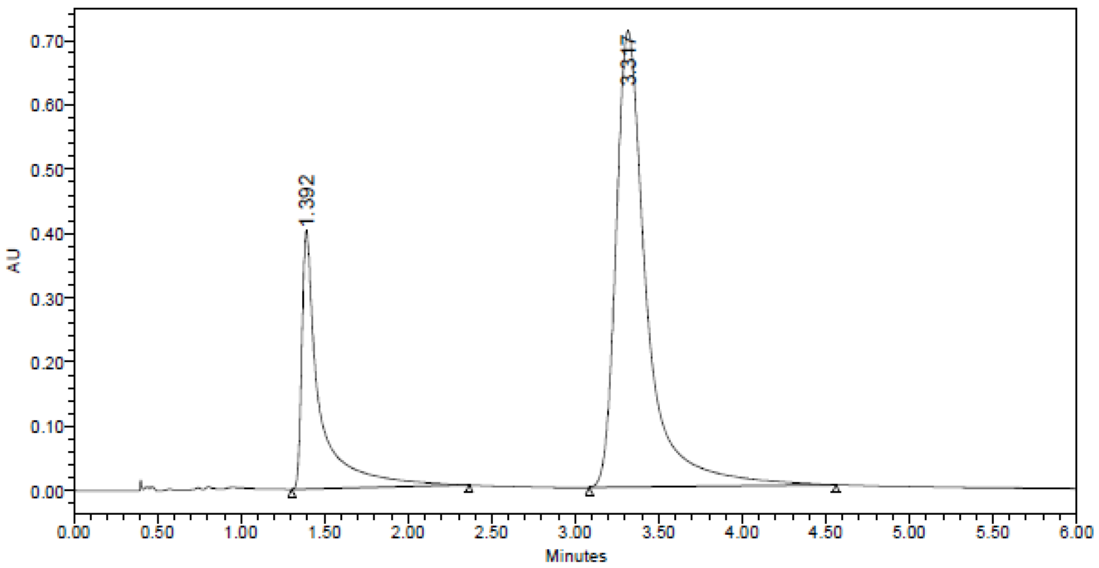

	RT	Area	% Area	Height
1	1.392	3012863	24.90	401145
2	3.317	9088267	75.10	708773

The chiral HPLC chromatogram of **7**

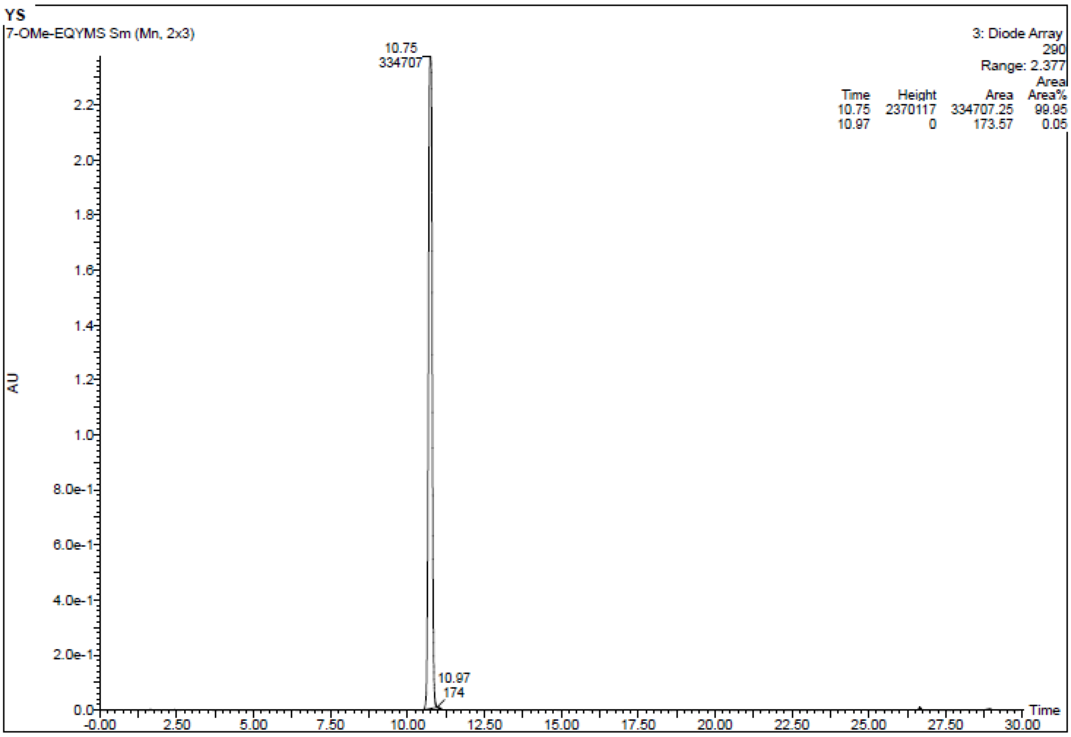

The purity of compound **7** at 290 nm

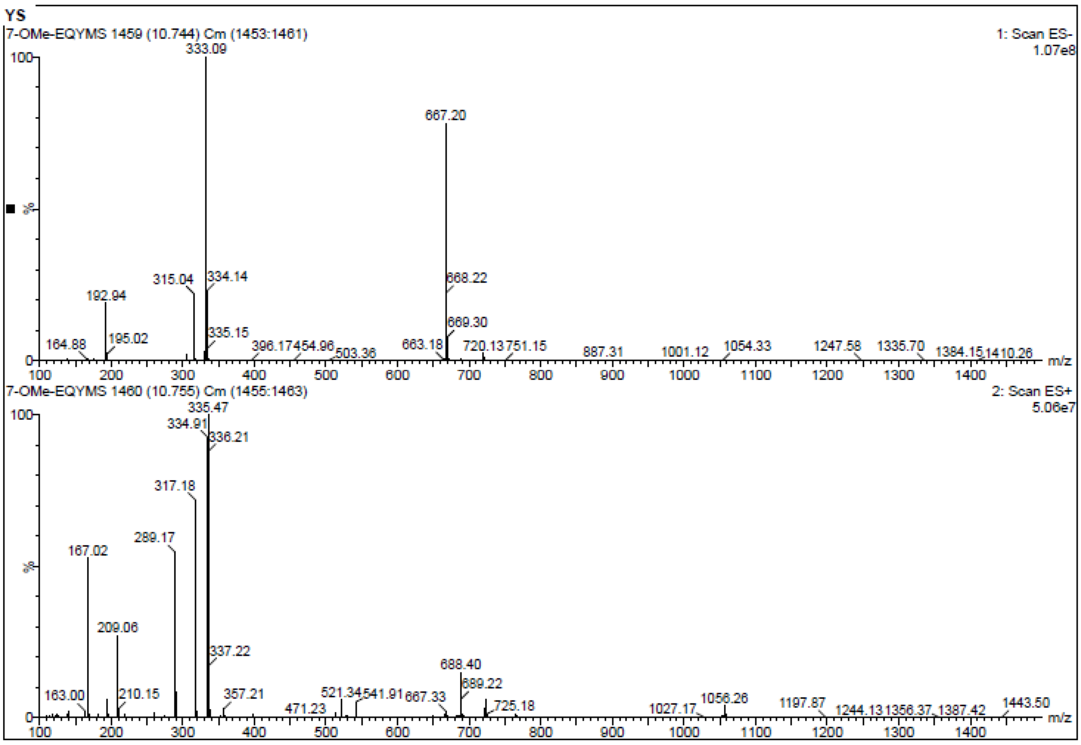

ESI-MS spectrum of compound **7**

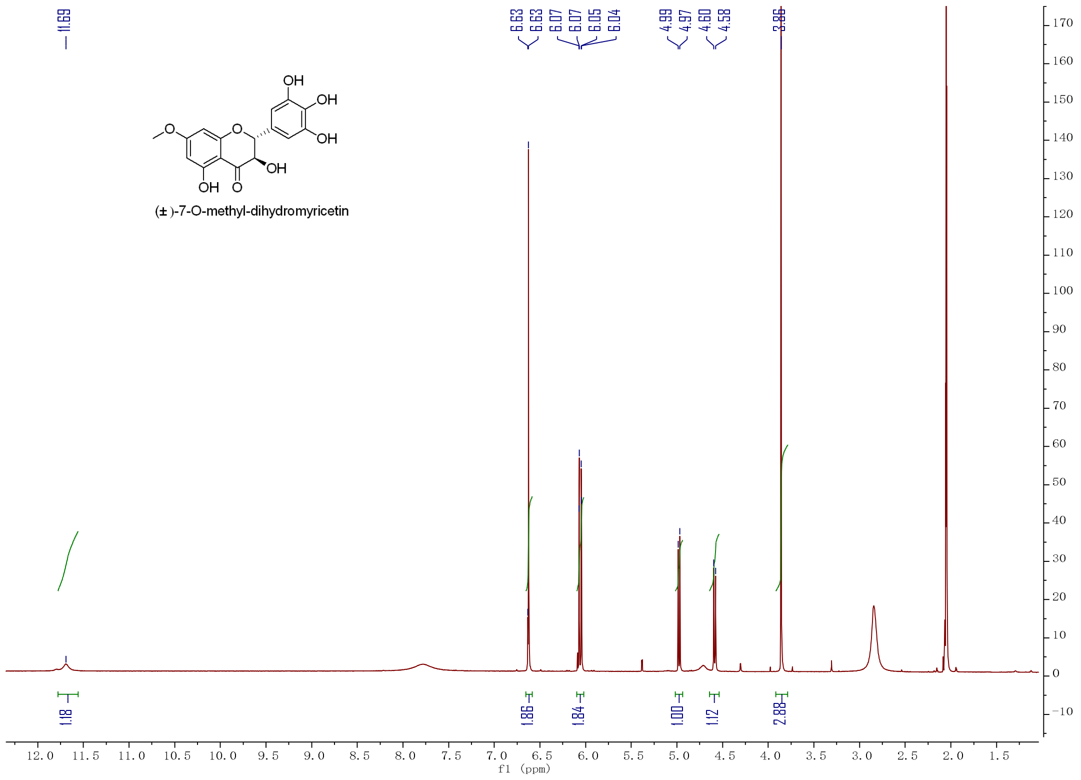

^{1}H NMR (Acetone-d_6, 600 MHz) spectrum of compound **7**

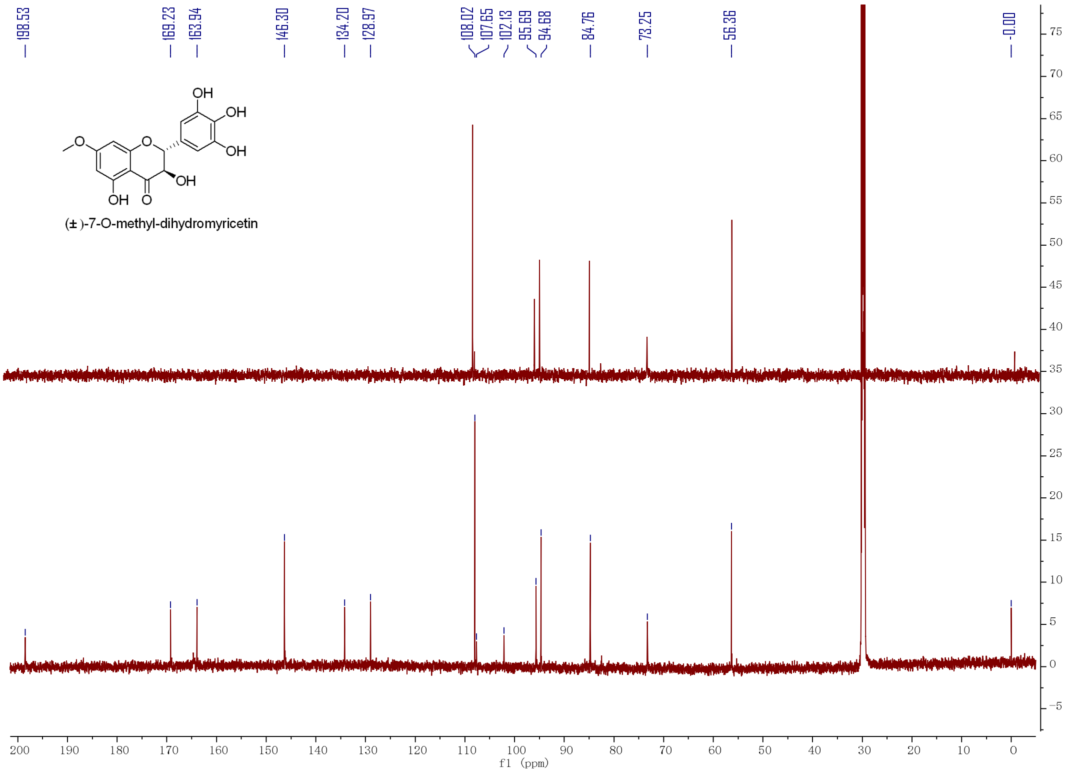

^{13}C NMR (Acetone-d_6, 150 MHz) and DEPT-135 spectra of compound **7**

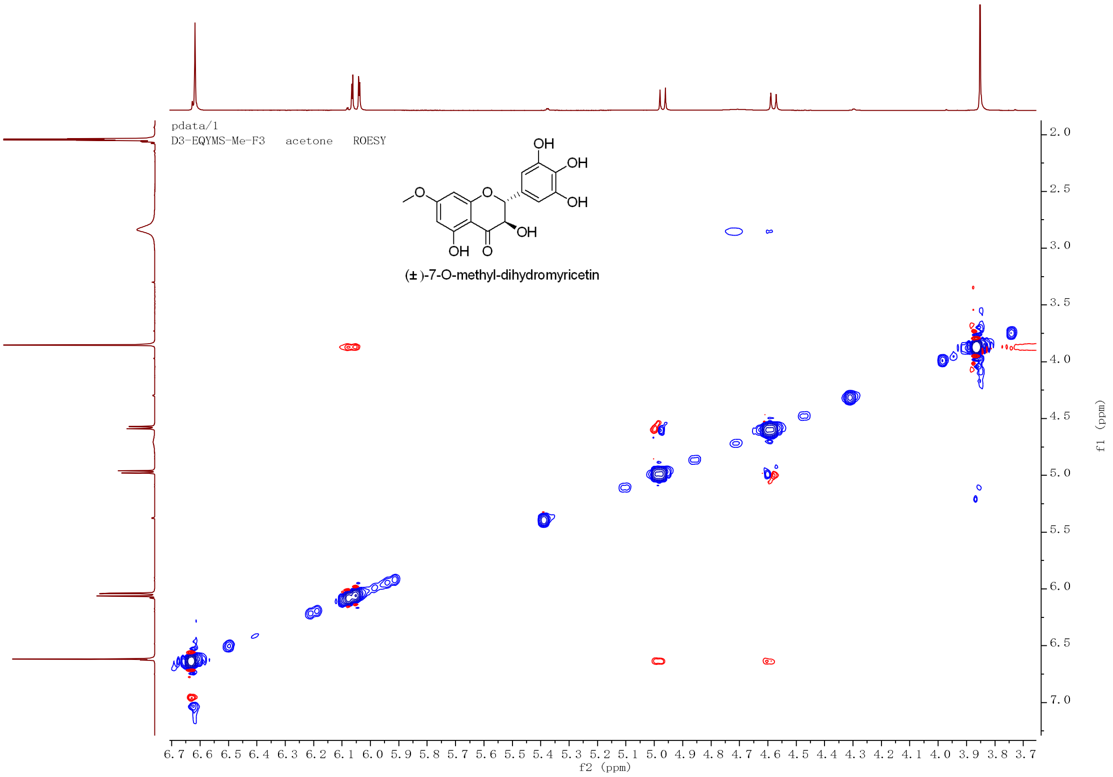

ROSEY spectrum of compound **7**

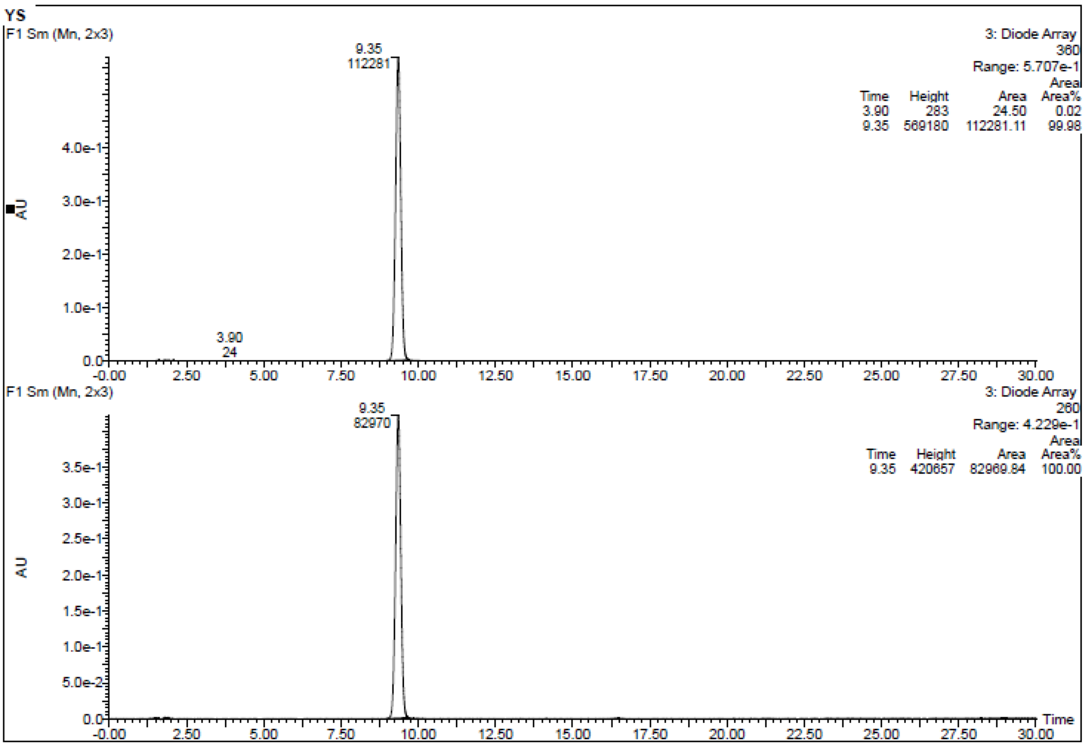

The purity of compound **8** at 360 nm and 260 nm

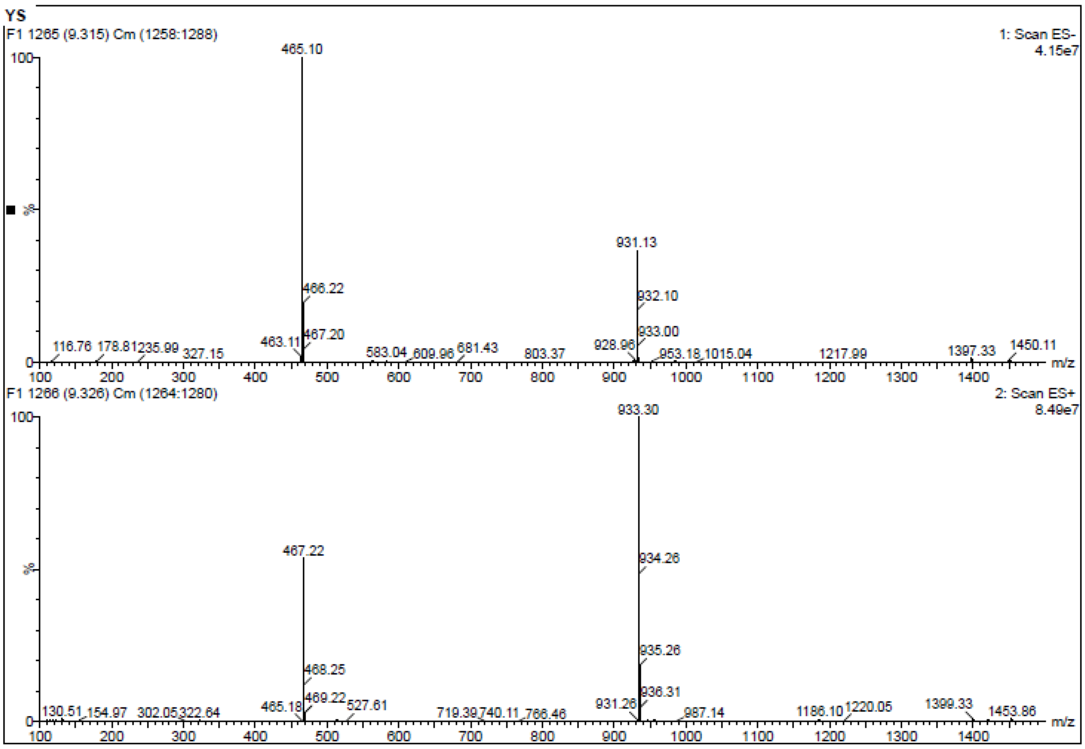

ESI-MS spectrum of compound **8**

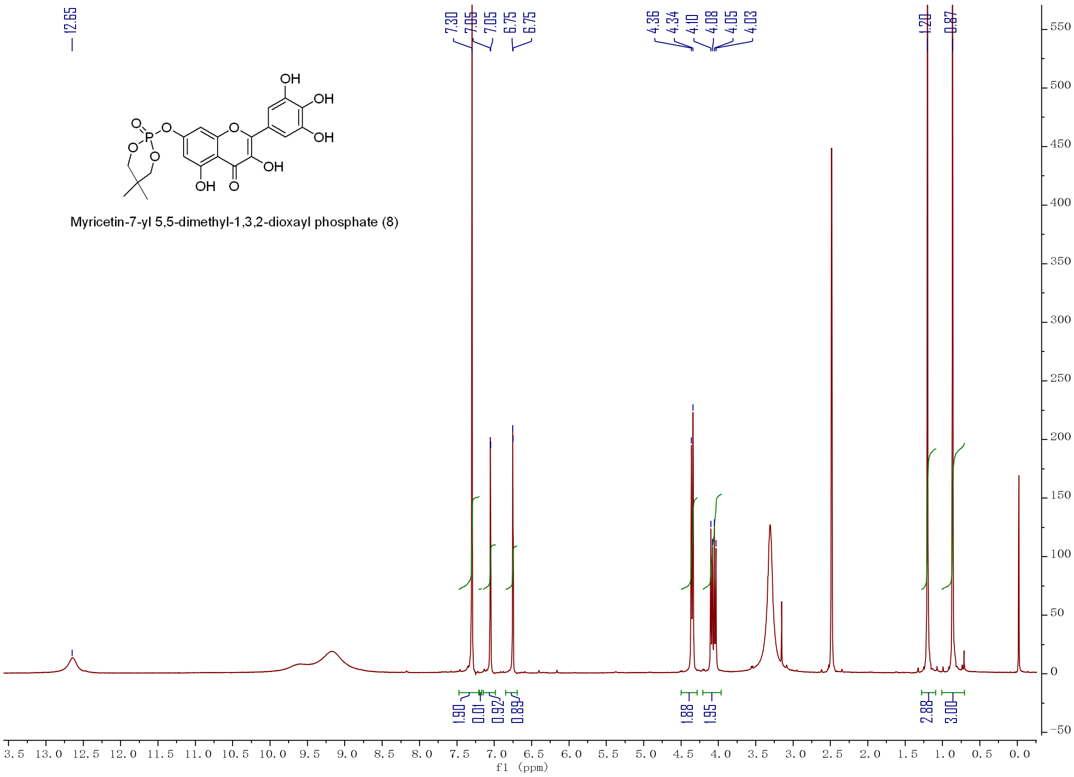

^{1}H NMR (DMSO-d_6, 500 MHz) spectrum of compound **8**

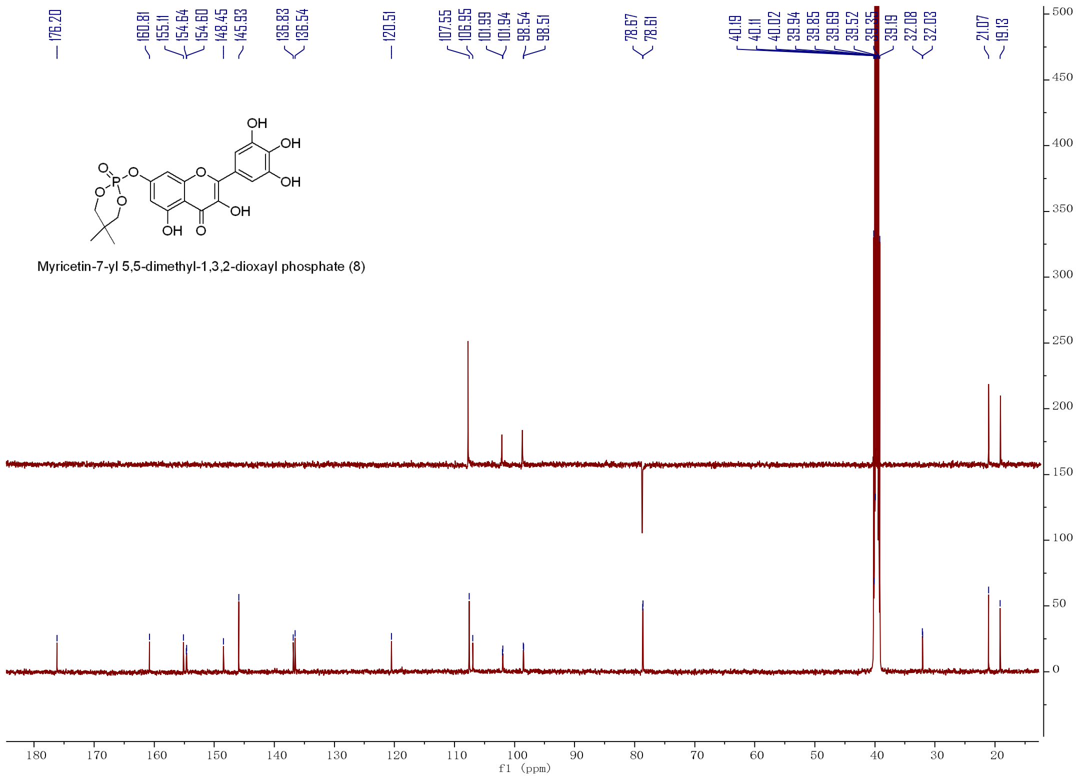

^{13}C NMR (DMSO-d_6, 125 MHz) and DEPT-135 spectra of compound **8**

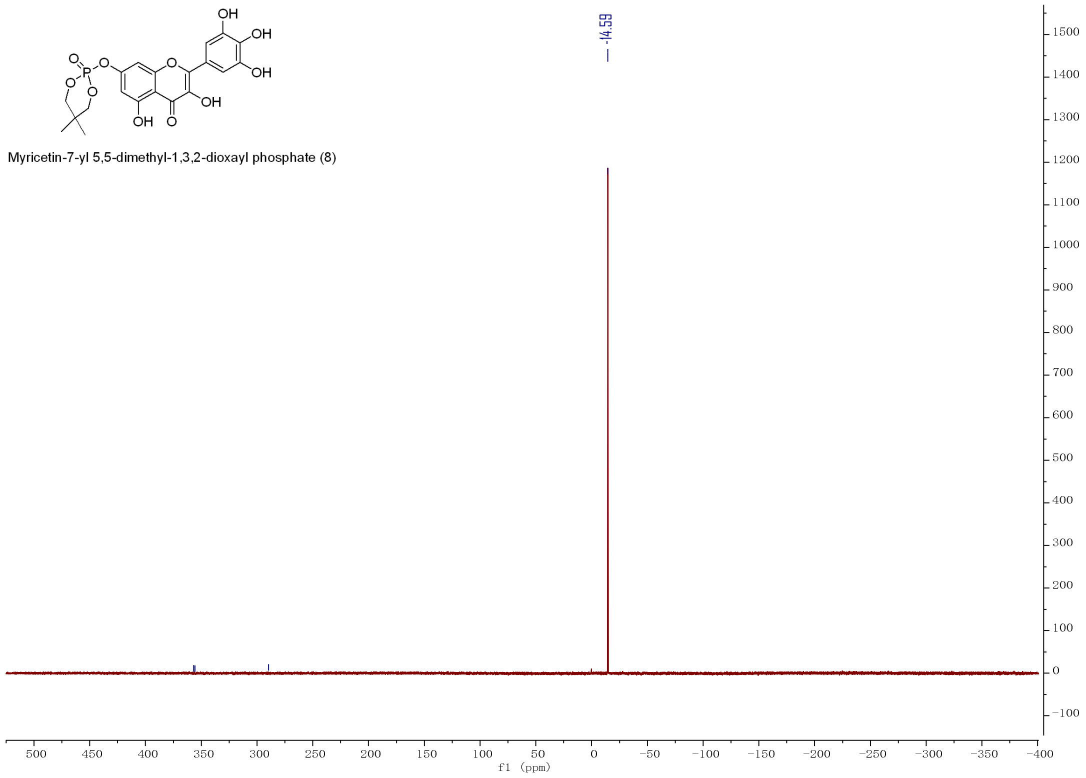

^{31}P NMR (DMSO-d_6, 125 MHz) spectrum of compound **8**

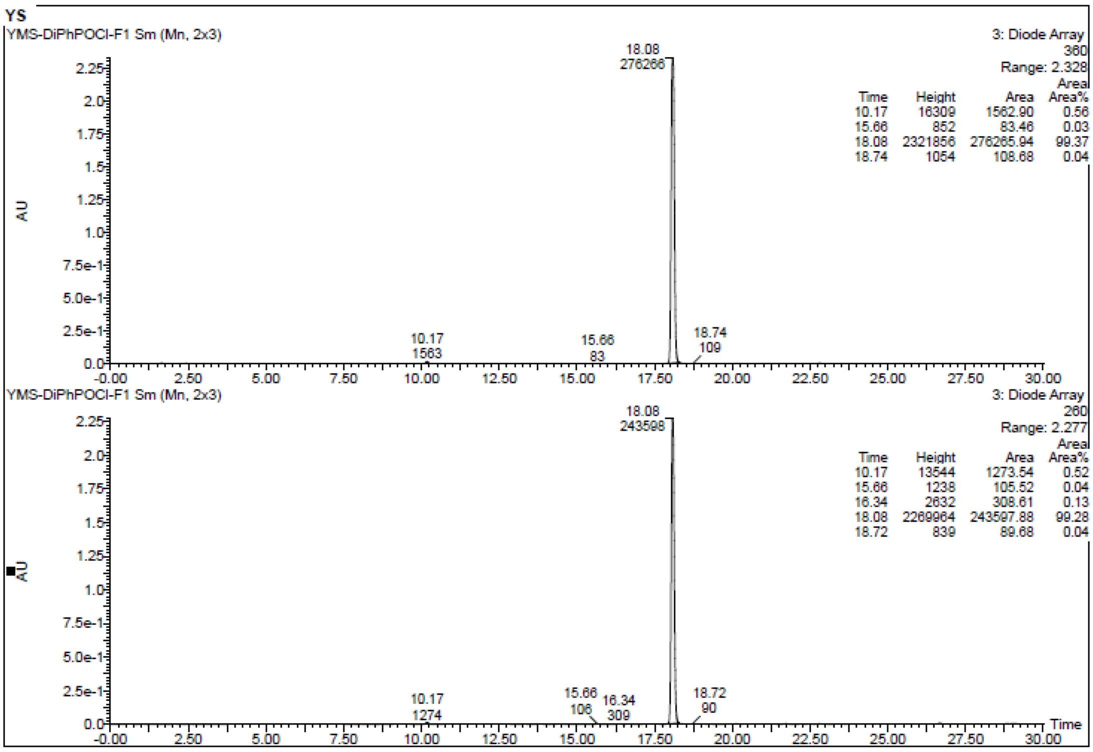

The purity of compound **9** at 360 nm and 260 nm

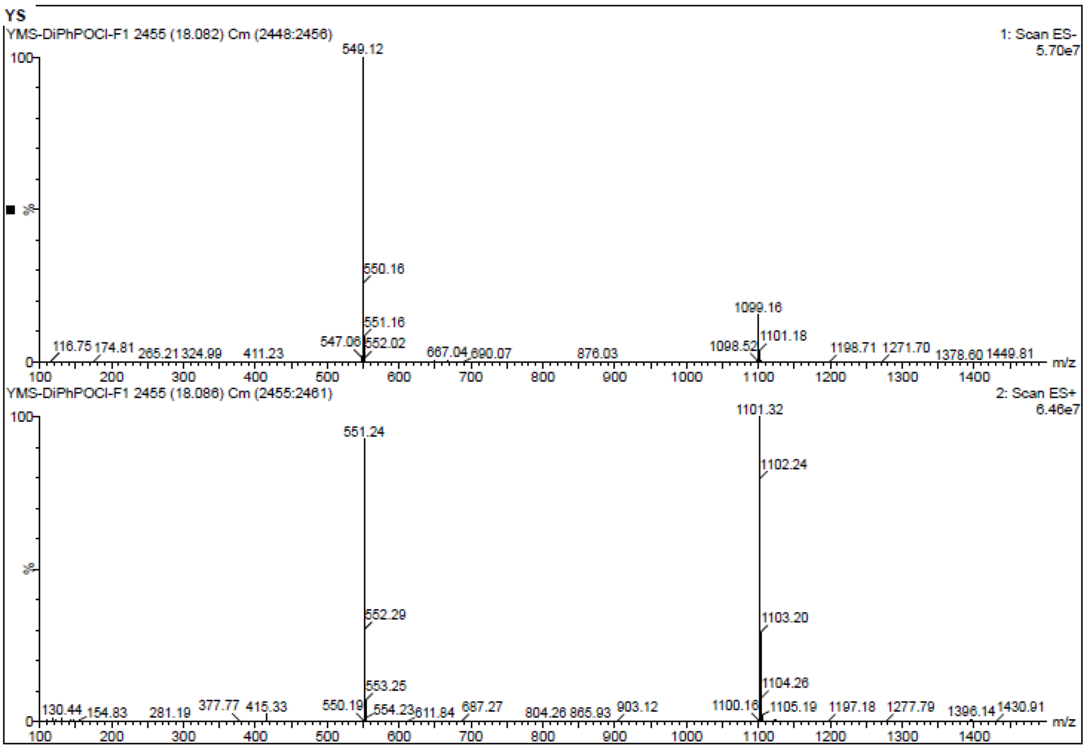

ESI-MS spectrum of compound **9**

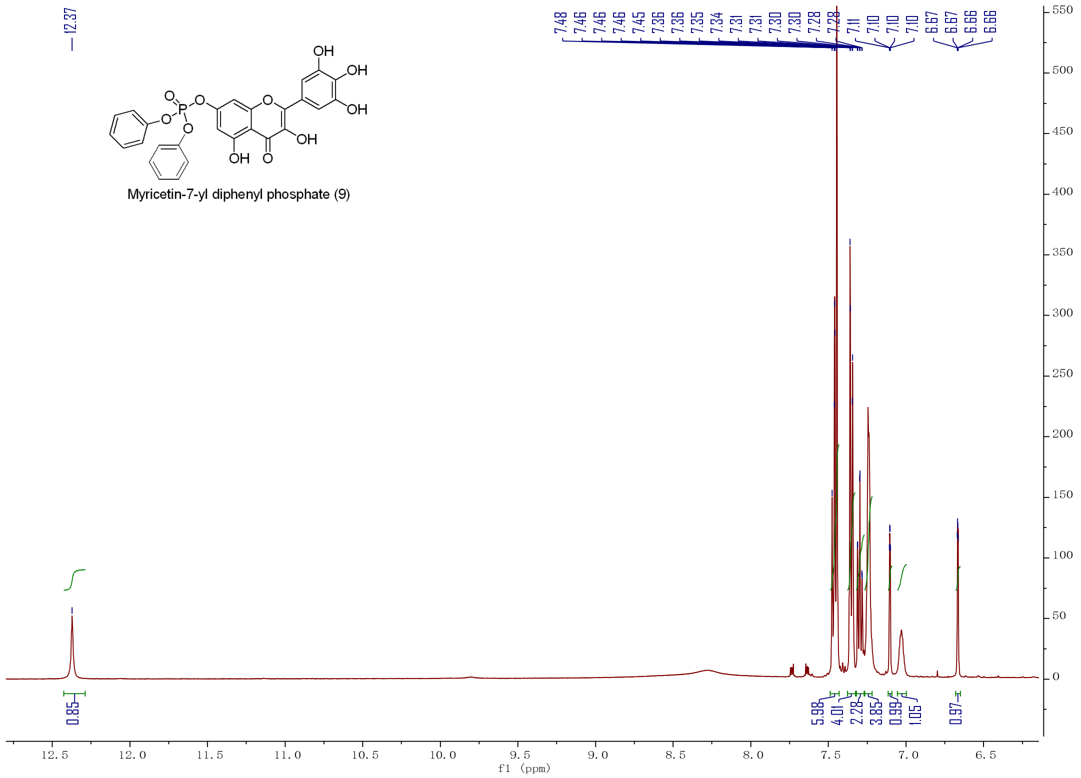

^{1}H NMR (Acetone-d_6, 500 MHz) spectrum of compound **9**

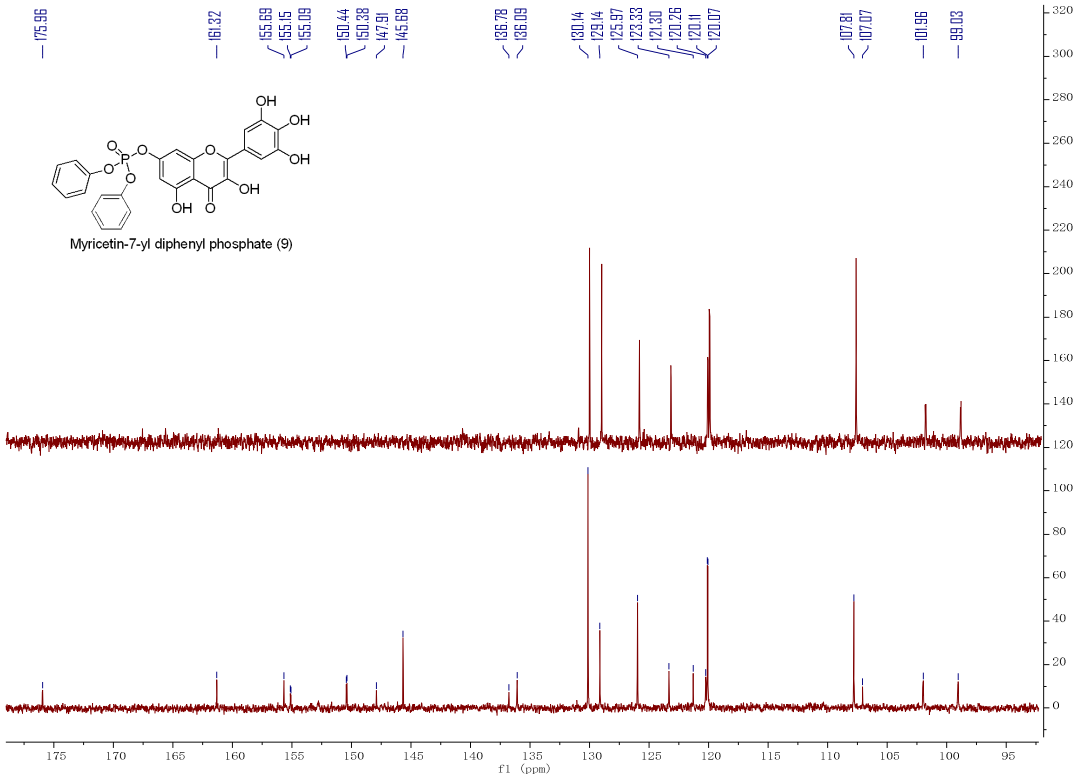

^{13}C NMR (Acetone-d_6, 125 MHz) and DEPT-135 spectra of compound **9**

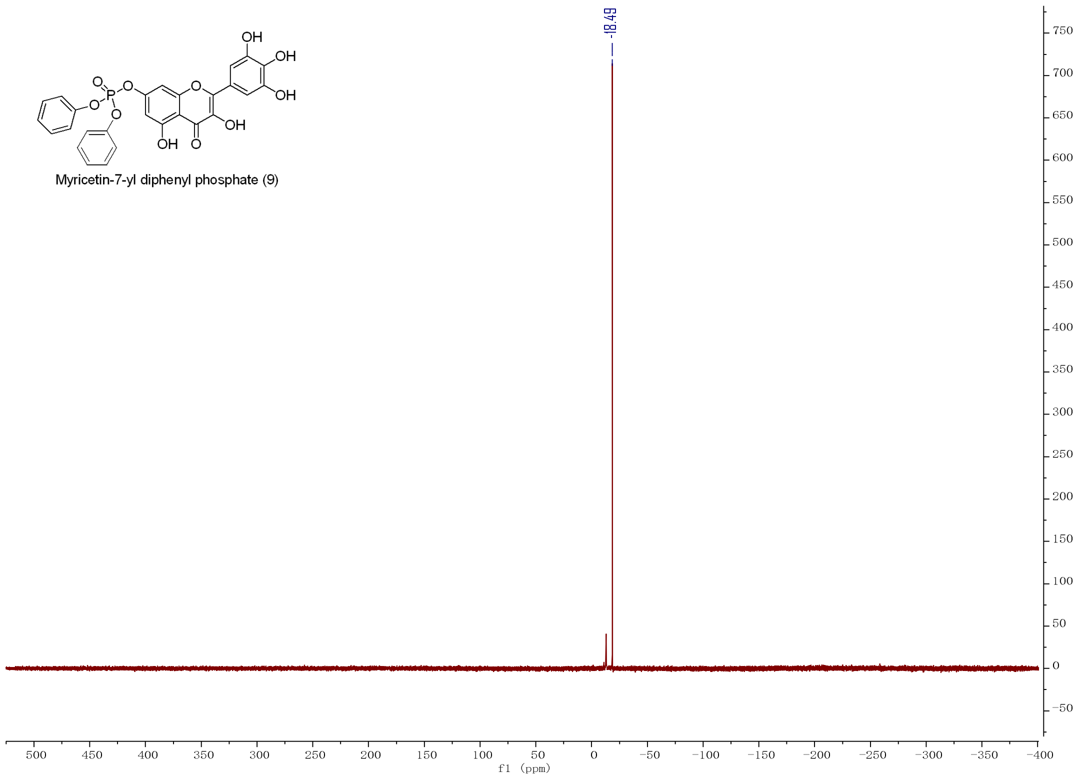

^{31}P NMR (Acetone-d_6, 125 MHz) spectrum of compound **9**

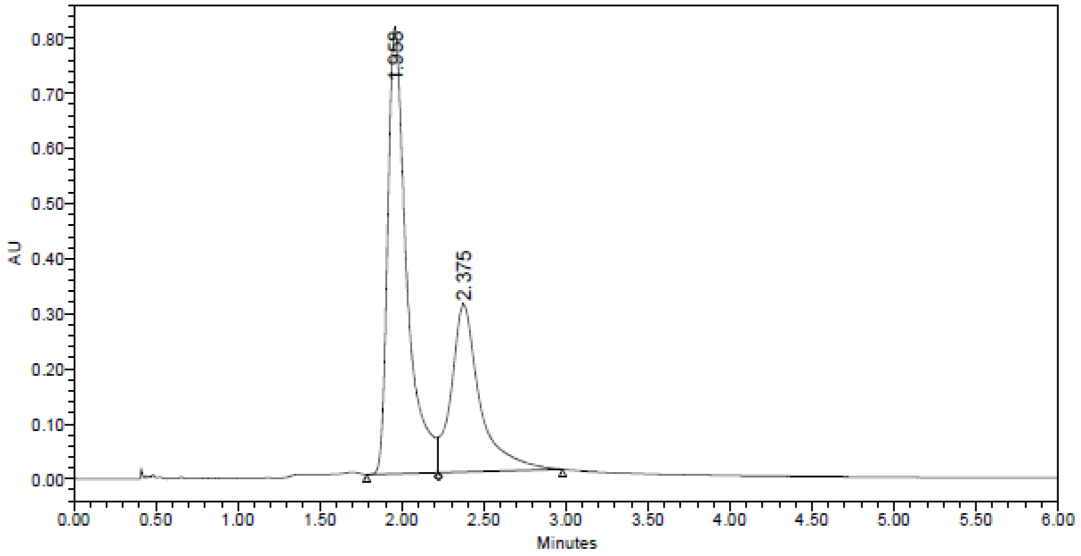

	RT	Area	% Area	Height
1	1.958	6533375	63.80	809835
2	2.375	3707484	36.20	303999

The chiral HPLC chromatogram of **10**

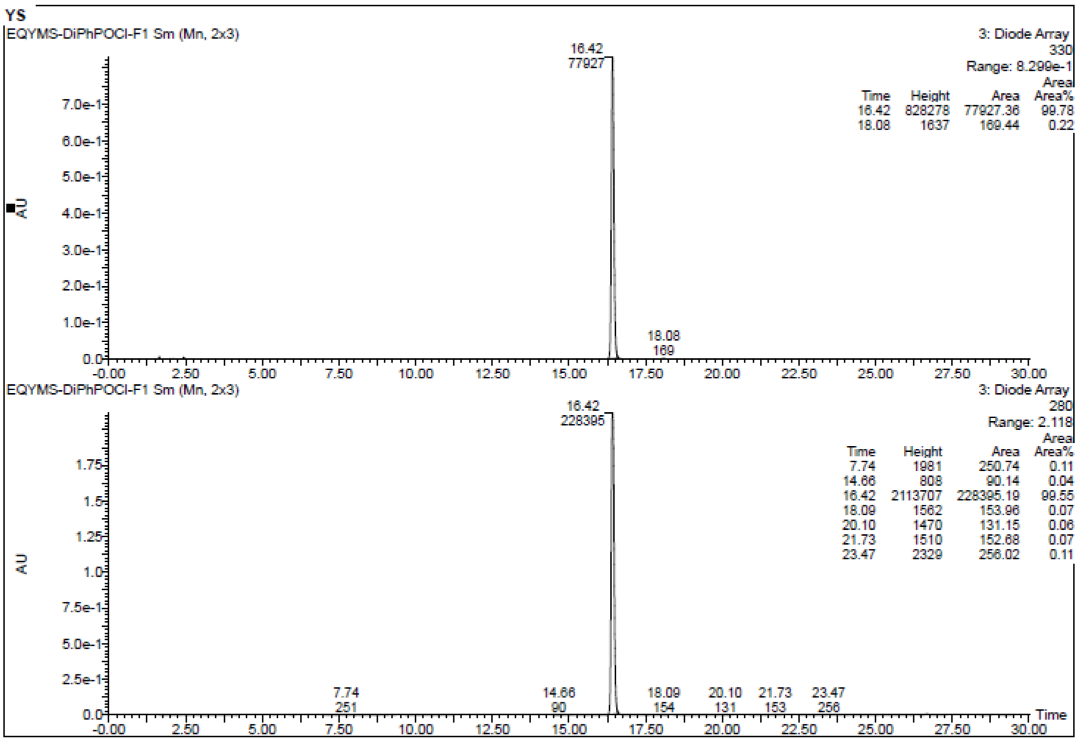

The purity of compound **10** at 330 nm and 280 nm

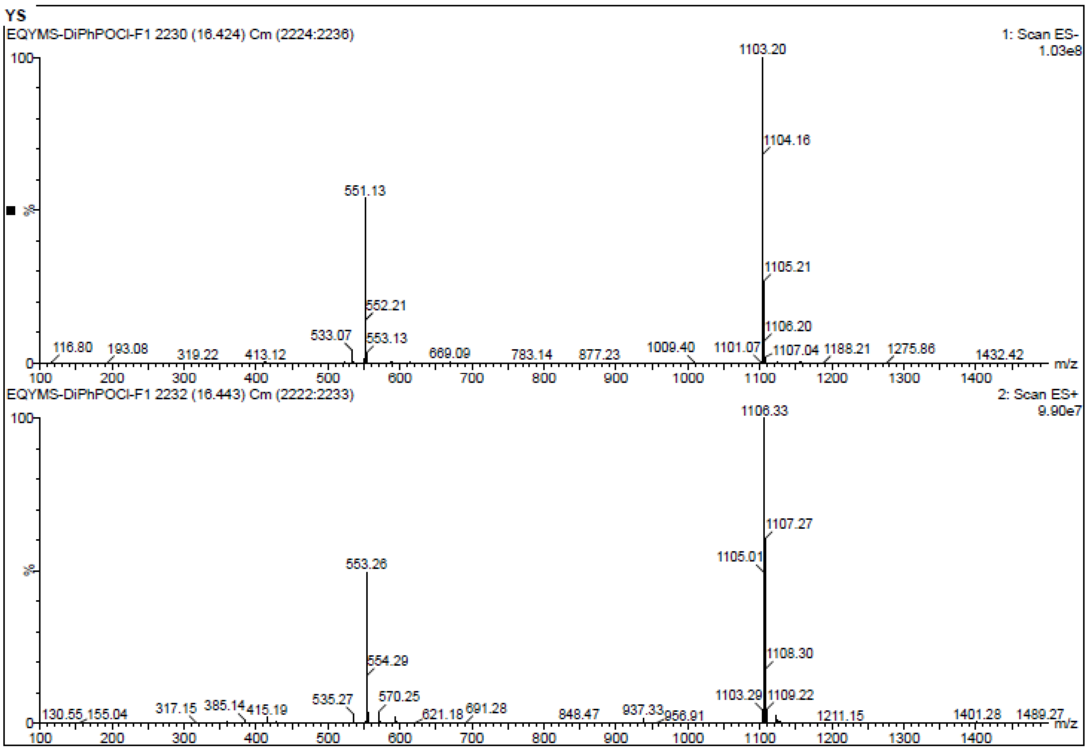

ESI-MS spectrum of compound **10**

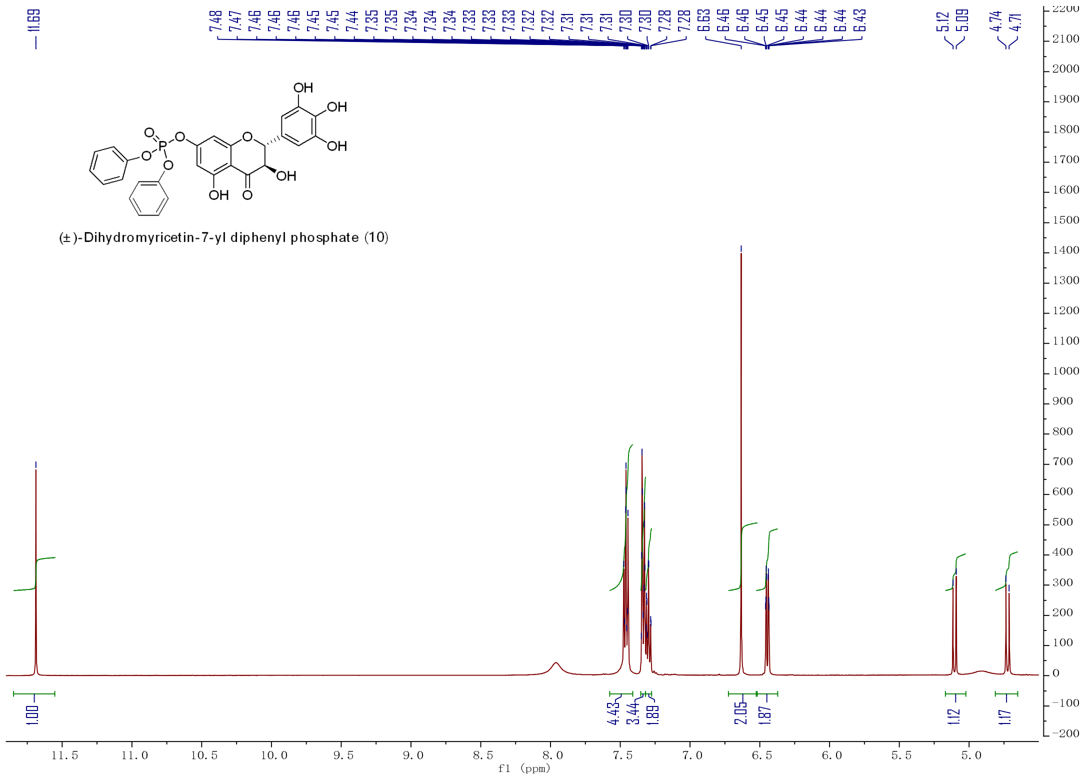

^1H NMR (Acetone-d_6, 500 MHz) spectrum of compound **10**

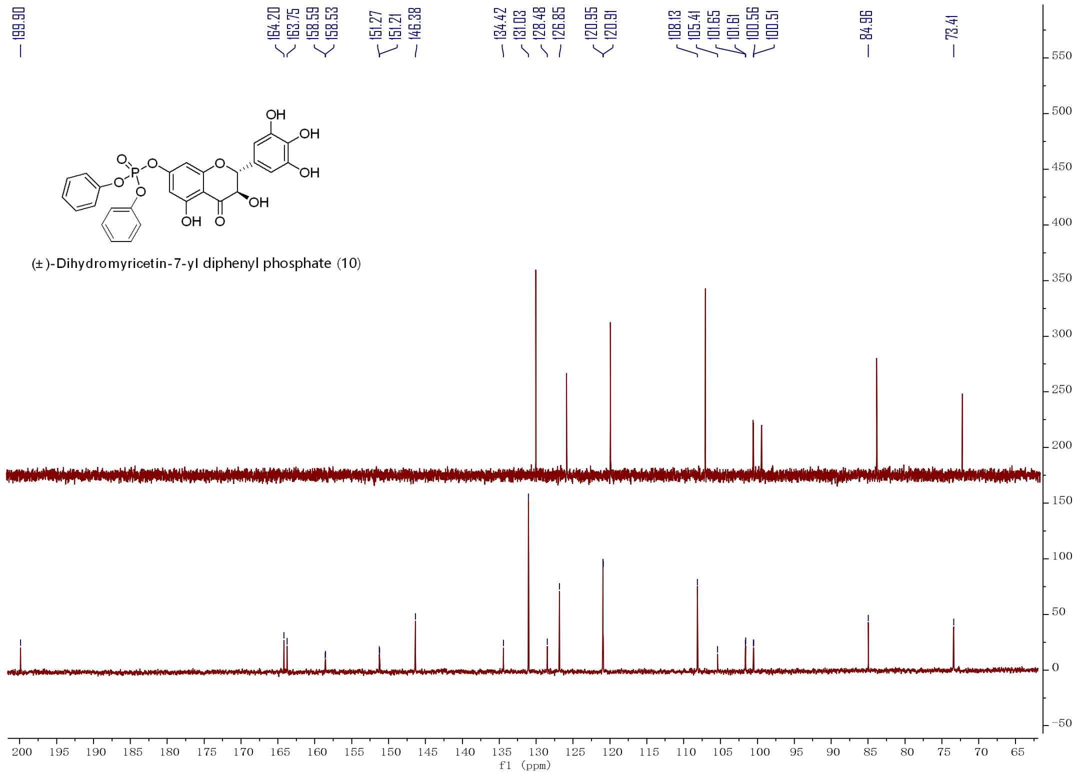

^{13}C NMR (Acetone-d_6, 125 MHz) and DEPT-135 spectra of compound **10**

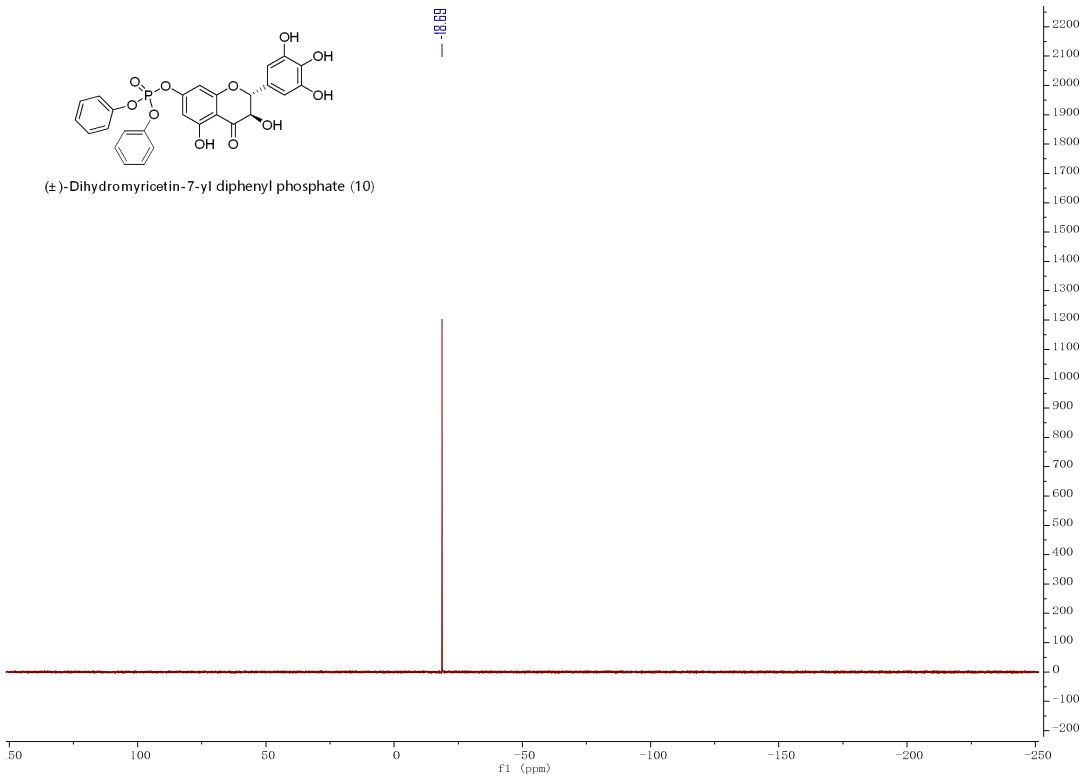

^{31}P NMR (Acetone-d_6, 125 MHz) spectrum of compound **10**

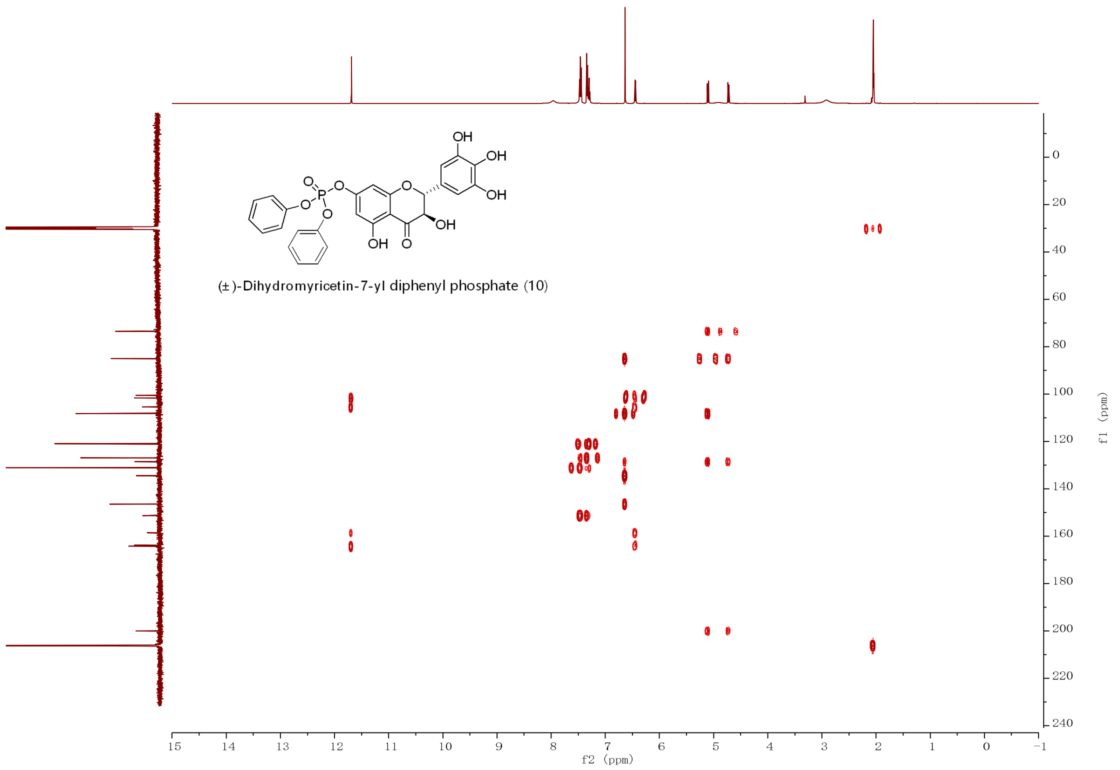

HMBC spectrum of compound **10**

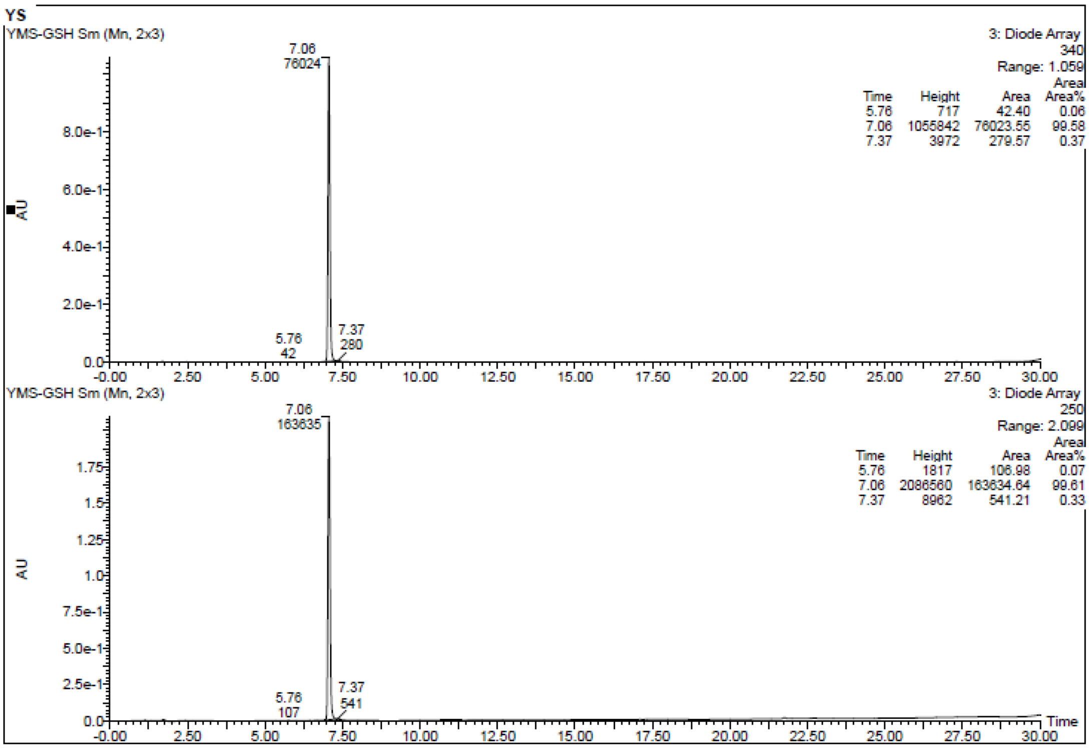

The purity of myricetin-GSH adduct at 330 nm and 280 nm

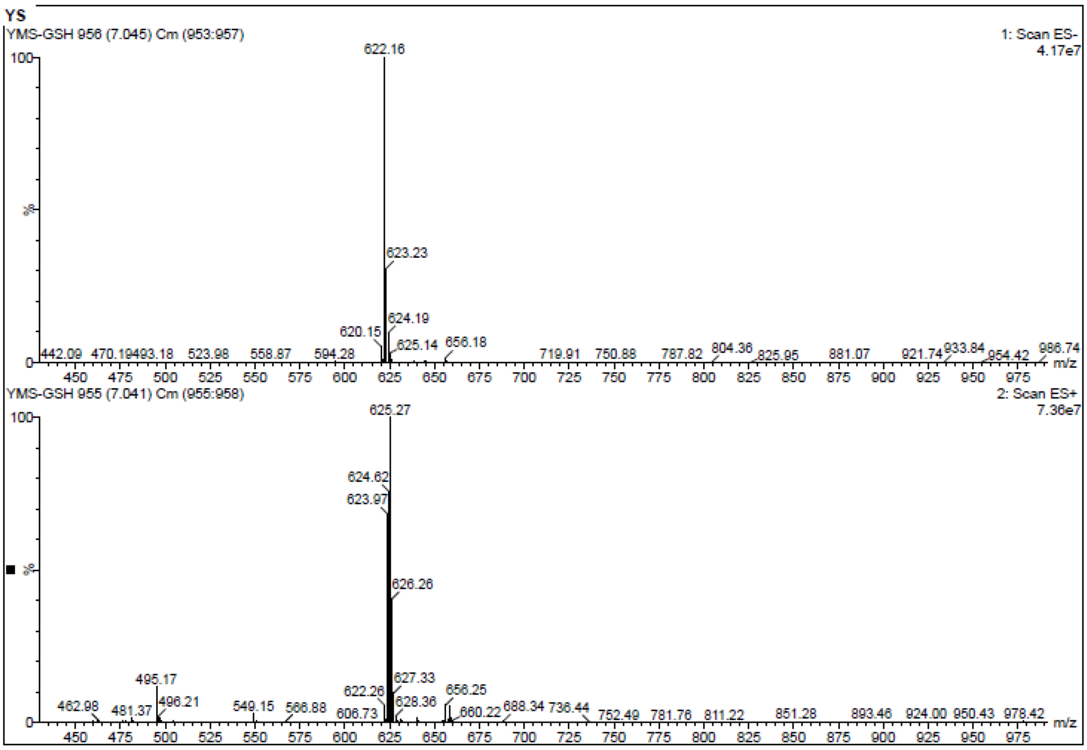

ESI-MS spectrum of myricetin-GSH adduct

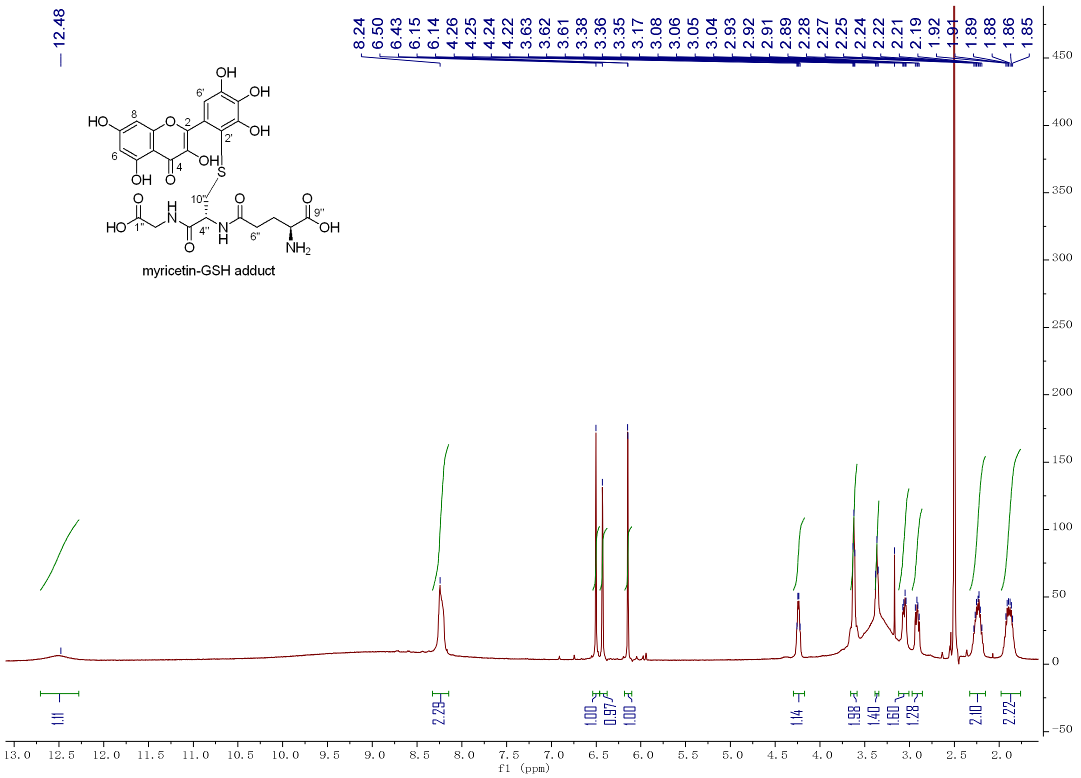

^{1}H NMR (DMSO-d_6, 500 MHz) spectrum of myricetin-GSH adduct

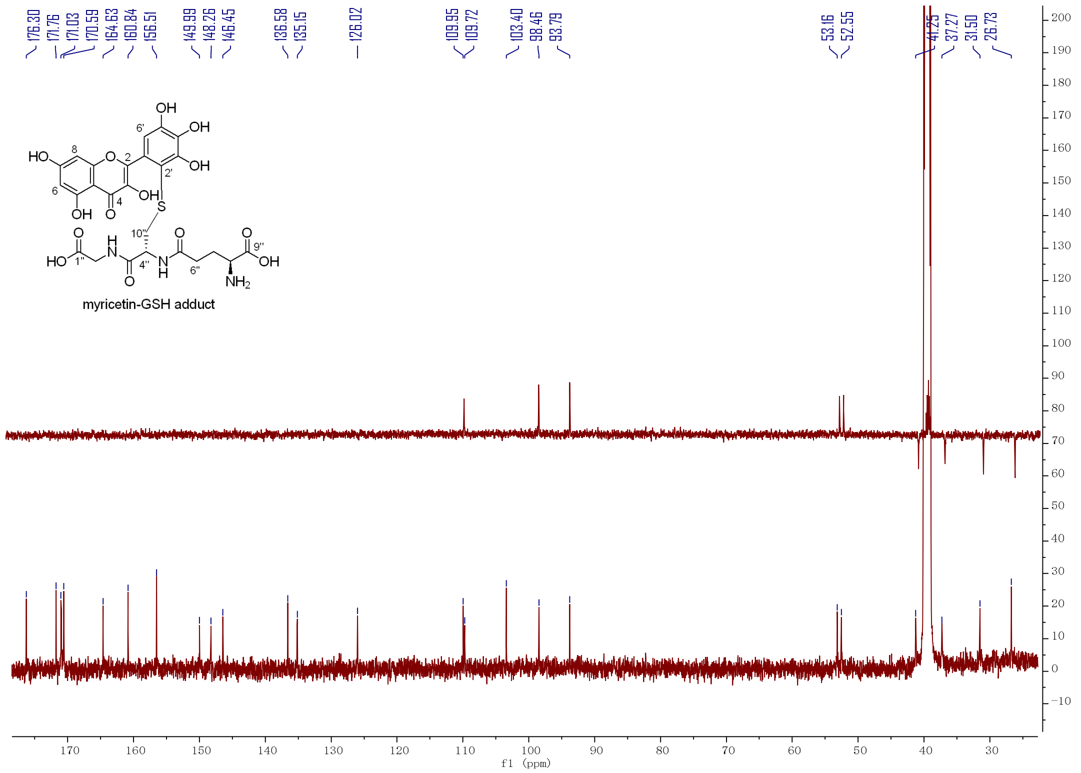

^{13}C NMR (Acetone-d_6, 125 MHz) and DEPT-135 spectra of myricetin-GSH adduct
