# Supplementary material for: Identification of pyrogallol as a warhead in design of covalent inhibitors for the SARS-CoV-2 3CL protease
Source: Nat Commun. 2021 Jun 15;12:3623. doi: 10.1038/s41467-021-23751-3 (PMC8206078; doi:10.1038/s41467-021-23751-3)
Supplement: Supplementary file 3 — Reporting summary [file 41467_2021_23751_MOESM3_ESM.pdf]

nature research

Corresponding author(s): Yechun Xu

Last updated by author(s): May 13, 2021

Reporting Summary

Nature Research wishes to improve the reproducibility of the work that we publish. This form provides structure for consistency and transparency in reporting. For further information on Nature Research policies, see our Editorial Policies and the Editorial Policy Checklist.

Statistics

For all statistical analyses, confirm that the following items are present in the figure legend, table legend, main text, or Methods section.

n/a	Confirmed	
☐	☒	The exact sample size (*n*) for each experimental group/condition, given as a discrete number and unit of measurement
☐	☒	A statement on whether measurements were taken from distinct samples or whether the same sample was measured repeatedly
☒	☐	The statistical test(s) used AND whether they are one- or two-sided<br>*Only common tests should be described solely by name; describe more complex techniques in the Methods section.*
☒	☐	A description of all covariates tested
☒	☐	A description of any assumptions or corrections, such as tests of normality and adjustment for multiple comparisons
☐	☒	A full description of the statistical parameters including central tendency (e.g. means) or other basic estimates (e.g. regression coefficient) AND variation (e.g. standard deviation) or associated estimates of uncertainty (e.g. confidence intervals)
☒	☐	For null hypothesis testing, the test statistic (e.g. *F*, *t*, *r*) with confidence intervals, effect sizes, degrees of freedom and *P* value noted<br>*Give P values as exact values whenever suitable.*
☒	☐	For Bayesian analysis, information on the choice of priors and Markov chain Monte Carlo settings
☒	☐	For hierarchical and complex designs, identification of the appropriate level for tests and full reporting of outcomes
☒	☐	Estimates of effect sizes (e.g. Cohen's *d*, Pearson's *r*), indicating how they were calculated

Our web collection on statistics for biologists contains articles on many of the points above.

Software and code

Policy information about availability of computer code

Data collection	Bluice 5.0 was used to collect X-ray diffraction data;<br>NMR data were recorded on a Bruker AVANCE III 600 MHz instrument;<br>ESIMS spectra were collected on a Waters 2695 instrument with a 2998 PDA detector coupled with a Waters Acquity ELSD and a Waters 3100 SQDMS detector;<br>HRESIMS data were recorded on a Waters Synapt G2-Si Q-Tof mass detector;<br>Concentrations of remaining myricetin and myricetin-GSH adduct were determined on a SCIEX Triple QuadTM 5500 mass spectrometer (AB SCIEX, Concord, Ontario, Canada) coupled with Waters AcquityTM I-class system (Waters Corp, Milford, MA, USA);<br>In-gel fluorescence scanning was carried out by Typhoon FLA 9500 (GE Healthcare);<br>Plasma concentrations of the compounds were collected using the LC-MS/MS (an AQUITY UPLC system with a thermostatted autosampler, an ultrahigh performance binary pump (I-class, Waters, MA, USA), and a triple quadrupole mass spectrometer with electrospray ionization (ESI) source (Xevo TQ-S, Waters, MA, USA);<br>AMBER 18 suite of program was employed for simulation with the underlying force fields of FF99SBildn force field for protein and TIP3P model for water.

Data analysis	Graphpad Prism software 8.0 was used to determine the IC50 values and EC50 values;<br>For the analysis of crystallography data, we used HKL3000, Phaser 2.7.0, COOT 0.8.8, PHENIX 1.11.1-2575, Pymol 2.0.6;<br>Masslynx V4.1 SCN 805 was used to analyze the ESIMS data for all compounds;<br>NMR data for all compounds was performed on MestReNova x64-12.0.0-20080;<br>Quantitative analysis for half-life determination of myricetin reacting with glutathione was performed on Analyst 1.6.3 software (AB SCIEX, Concord, Ontario, Canada);

For manuscripts utilizing custom algorithms or software that are central to the research but not yet described in published literature, software must be made available to editors and reviewers. We strongly encourage code deposition in a community repository (e.g. GitHub). See the Nature Research guidelines for submitting code & software for further information.

Data

Policy information about availability of data

All manuscripts must include a data availability statement. This statement should provide the following information, where applicable:

- Accession codes, unique identifiers, or web links for publicly available datasets
- A list of figures that have associated raw data
- A description of any restrictions on data availability

The atomic coordinates and structure factors have been deposited into the Protein Data Bank with accession codes 7DPP [https://doi.org/10.2210/pdb7DPP/pdb] (SARS-CoV-2 3CLpro in complex with myricetin), 7DPU [https://doi.org/10.2210/pdb7DPU/pdb] (SARS-CoV-2 3CLpro in complex with 3), and 7DPV [https://doi.org/10.2210/pdb7DPV/pdb] (SARS-CoV-2 3CLpro in complex with 7).
A source data file is provided with the associated raw data of Fig. 1b, c; Fig. 3a, c-f; Supplementary Fig. 2-5, 11; and Supplementary Tables 4. All data are available from the corresponding author upon reasonable request.
The cDNA of SARS-CoV-2 3CLpro and PLpro (GenBank: MN908947.3, https://https.ncbi.nlm.nih.gov/nuccore/MN908947.3) or SARS-CoV 3CLpro (GenBank: AAP13442.1, https://https.ncbi.nlm.nih.gov/protein/AAP13442.1) were obtained from Genbank (https://https.ncbi.nlm.nih.gov/genbank/).

Field-specific reporting

Please select the one below that is the best fit for your research. If you are not sure, read the appropriate sections before making your selection.

[x] Life sciences [ ] Behavioural & social sciences [ ] Ecological, evolutionary & environmental sciences

For a reference copy of the document with all sections, see nature.com/documents/nr-reporting-summary-flat.pdf

Life sciences study design

All studies must disclose on these points even when the disclosure is negative.

Sample size	Sample sizes are indicated in the figure legends. A sample size of at least three independent biological replicates were chosen for all experiments , as is standard for biochemistry assays.
Data exclusions	No data were excluded from analyses.
Replication	All experiments had at least three independent replicates as indicated in the figure legends. All attempts at replication were successful.
Randomization	Randomization was not relevant to this study, beacause no grouping was needed.
Blinding	Blinding was not performed in this study based on the nature of structural biology. In biochemistry experiments, all data form experimental and control groups were used for statistical inference and drawing conclusions.

Reporting for specific materials, systems and methods

We require information from authors about some types of materials, experimental systems and methods used in many studies. Here, indicate whether each material, system or method listed is relevant to your study. If you are not sure if a list item applies to your research, read the appropriate section before selecting a response.

Materials & experimental systems

n/a	Involved in the study	
x		Antibodies
	x	Eukaryotic cell lines
x		Palaeontology and archaeology
	x	Animals and other organisms
x		Human research participants
x		Clinical data
x		Dual use research of concern

Methods

n/a	Involved in the study	
x		ChIP-seq
x		Flow cytometry
x		MRI-based neuroimaging

Eukaryotic cell lines

Policy information about cell lines

Cell line source(s)	The Vero E6 and HEK293T cell lines were purchased from American Type Culture Collection (ATCC, Manassas, USA).
Authentication	The cell lines were not authenticated.
Mycoplasma contamination	The cells was tested negative for mycoplasma contamination.
Commonly misidentified lines (See ICLAC register)	No commonly misidentified lines were used.

Animals and other organisms

Policy information about studies involving animals; ARRIVE guidelines recommended for reporting animal research

Laboratory animals	Six-week-old ICR male mice were housed in a 12/12-h light/dark cycles at 25℃ and humidity 40-70% with regular chow diet and free access to water.
Wild animals	This study did not involve the wild animals.
Field-collected samples	This study did not involve samples collected from the field.
Ethics oversight	All animal experiments were performed following animal ethics guidelines and protocols approved by the Institutional Animal Care and Use Committee of Shanghai Institute of Materia Medica (Accreditation number: 2020-02-YY-11).

Note that full information on the approval of the study protocol must also be provided in the manuscript.
